# deGPS is a powerful tool for detecting differential expression in RNA-sequencing studies

**DOI:** 10.1186/s12864-015-1676-0

**Published:** 2015-06-13

**Authors:** Chen Chu, Zhaoben Fang, Xing Hua, Yaning Yang, Enguo Chen, Allen W. Cowley, Mingyu Liang, Pengyuan Liu, Yan Lu

**Affiliations:** Department of Statistics and Finance, University of Science and Technology of China, Hefei, Anhui 230026 China; Department of Physiology, Medical College of Wisconsin, Milwaukee, WI 53226 USA; Department of Gynecologic Oncology, The Affiliated Women’s Hospital, School of Medicine, Zhejiang University, Hangzhou, Zhejiang 310029 China; Division of Respiratory Medicine, Sir Run Run Shaw Hospital, School of Medicine, Zhejiang University, Hangzhou, Zhejiang 310058 China; Institute for Translational Medicine, School of Medicine, Zhejiang University, Hangzhou, Zhejiang 310029 China

**Keywords:** Next-generation sequencing, Differential expression, Generalized Poisson, RNA-Seq

## Abstract

**Background:**

The advent of the NGS technologies has permitted profiling of whole-genome transcriptomes (i.e., RNA-Seq) at unprecedented speed and very low cost. RNA-Seq provides a far more precise measurement of transcript levels and their isoforms compared to other methods such as microarrays. A fundamental goal of RNA-Seq is to better identify expression changes between different biological or disease conditions. However, existing methods for detecting differential expression from RNA-Seq count data have not been comprehensively evaluated in large-scale RNA-Seq datasets. Many of them suffer from inflation of type I error and failure in controlling false discovery rate especially in the presence of abnormal high sequence read counts in RNA-Seq experiments.

**Results:**

To address these challenges, we propose a powerful and robust tool, termed deGPS, for detecting differential expression in RNA-Seq data. This framework contains new normalization methods based on generalized Poisson distribution modeling sequence count data, followed by permutation-based differential expression tests. We systematically evaluated our new tool in simulated datasets from several large-scale TCGA RNA-Seq projects, unbiased benchmark data from compcodeR package, and real RNA-Seq data from the development transcriptome of Drosophila. deGPS can precisely control type I error and false discovery rate for the detection of differential expression and is robust in the presence of abnormal high sequence read counts in RNA-Seq experiments.

**Conclusions:**

Software implementing our deGPS was released within an R package with parallel computations (https://github.com/LL-LAB-MCW/deGPS). deGPS is a powerful and robust tool for data normalization and detecting different expression in RNA-Seq experiments. Beyond RNA-Seq, deGPS has the potential to significantly enhance future data analysis efforts from many other high-throughput platforms such as ChIP-Seq, MBD-Seq and RIP-Seq.

**Electronic supplementary material:**

The online version of this article (doi:10.1186/s12864-015-1676-0) contains supplementary material, which is available to authorized users.

## Background

Next-generation sequencing (NGS) technologies parallelize the sequencing processes and produce millions of short-read sequences concurrently. The advent of the NGS technologies has permitted profiling of whole-genome transcriptomes by RNA-Seq, at unprecedented speed and very low cost. RNA-Seq provides a far more precise measurement of transcript levels and their isoforms compared to other methods such as microarrays [1].

In RNA-Seq experiments, millions of short sequence reads are aligned to a reference genome and the number of reads that fall into a particular genomic region is recorded, as read count data. These regions of interest are annotated as microRNA (miRNA), small interfering RNAs (siRNA), long noncoding RNAs (lncRNA), or messenger RNA (mRNA) in the context of RNA-Seq experiment, here all referred to as transcripts. The read count is linearly related to the abundance of target transcripts [2]. A major objective of RNA-Seq is to better identify count-based expression changes between different biological or disease conditions. A major challenge in differential expression analysis in RNA-Seq data is the unexpectedly large variability of sequence count data among transcripts. The observed count data are integers ranging theoretically from zero to infinite. Furthermore, read counts observed at a particular transcript location are limited by the depth of sequencing coverage and are dependent on the relative abundance of other transcripts. This differs from microarray experiments, where probe intensities for measuring transcript expression are independent of each other [3].

These unique features contained in RNA-Seq data have motivated the development of a number of statistical methods for data normalization and differential expression (DE) detection. Typical approaches use Poisson or negative binomial (NB) distribution to model count-based expression data. The Poisson distribution is commonly applied to models resulting in counting processes. It has a single parameter, which is uniquely determined by its mean. An important property of the Poisson distribution is that the mean equals its variance. However, read counts show a large variability in RNA-Seq experiments, and their variance is often much larger than their mean [4]. This is called the overdispersion problem. When overdispersion exists, the resulting Poisson-based tests will lead to biased and misleading conclusions.

To address the overdispersion problem, several statistical methods including DESeq [5] and edgeR [6] have been developed to model count data with NB distribution. The NB model adds an extra term to the variance of Poisson model to account for overdispersion. There are some technical differences between DESeq and edgeR for estimating the variance parameter of NB distribution. For instance, edgeR assumes that mean and variance are related and thus allows for estimating a common dispersion parameter throughout the whole experiment, followed by estimating trended and tagwise dispersions. DESeq allows for a flexible, mean-dependent location estimation of the dispersion.

Another alternative to the Poisson distribution is the generalized Poisson (GP) distribution [7]. The GP distribution introduces an extra parameter to the usual Poisson distribution. This extra parameter induces a loss of homogeneity in the stochastic counting processes modeled by the distribution. Both the NB and GP distributions can address the overdispersion problem and fix the bias resulting from using standard Poisson models. With same first two moments, GP distribution has heavier tail than NB distribution while NB distribution has larger mass at zero [7]. It is commonly observed that RNA-Seq data carry excessive zeroes or small read counts and are censored due to potential mapping errors. The GP distribution appears to fit such sequence count data better than the NB distribution on small values.

There are also some other methods developed for finding DE in RNA-Seq studies, e.g., NBPSeq [8], TSPM [9], baySeq [10], EBSeq [11],NOISeq [12], SAMseq [13] ShrinkSeq [14] and PoissonSeq [15]. Many of them were comprehensively reviewed and evaluated for their performance for finding count-based DE in several recent studies [3,16].

Here, we propose a powerful normalization method based on GP distribution modeling sequence count data, followed by regular permutation-based DE tests of GP-normalized data. Through comprehensive simulations, our method shows improved results for DE expression, in terms of false discovery rate (FDR), and sensitivity and specificity, in RNA-Seq experiments.

## Results

### Overview of deGPS

**Fig. 1:**
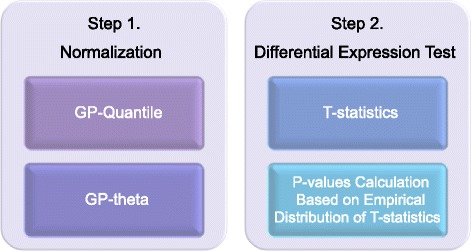
Overview of deGPS for analyzing sequence count data in RNA-Seq

**Fig. 2:**
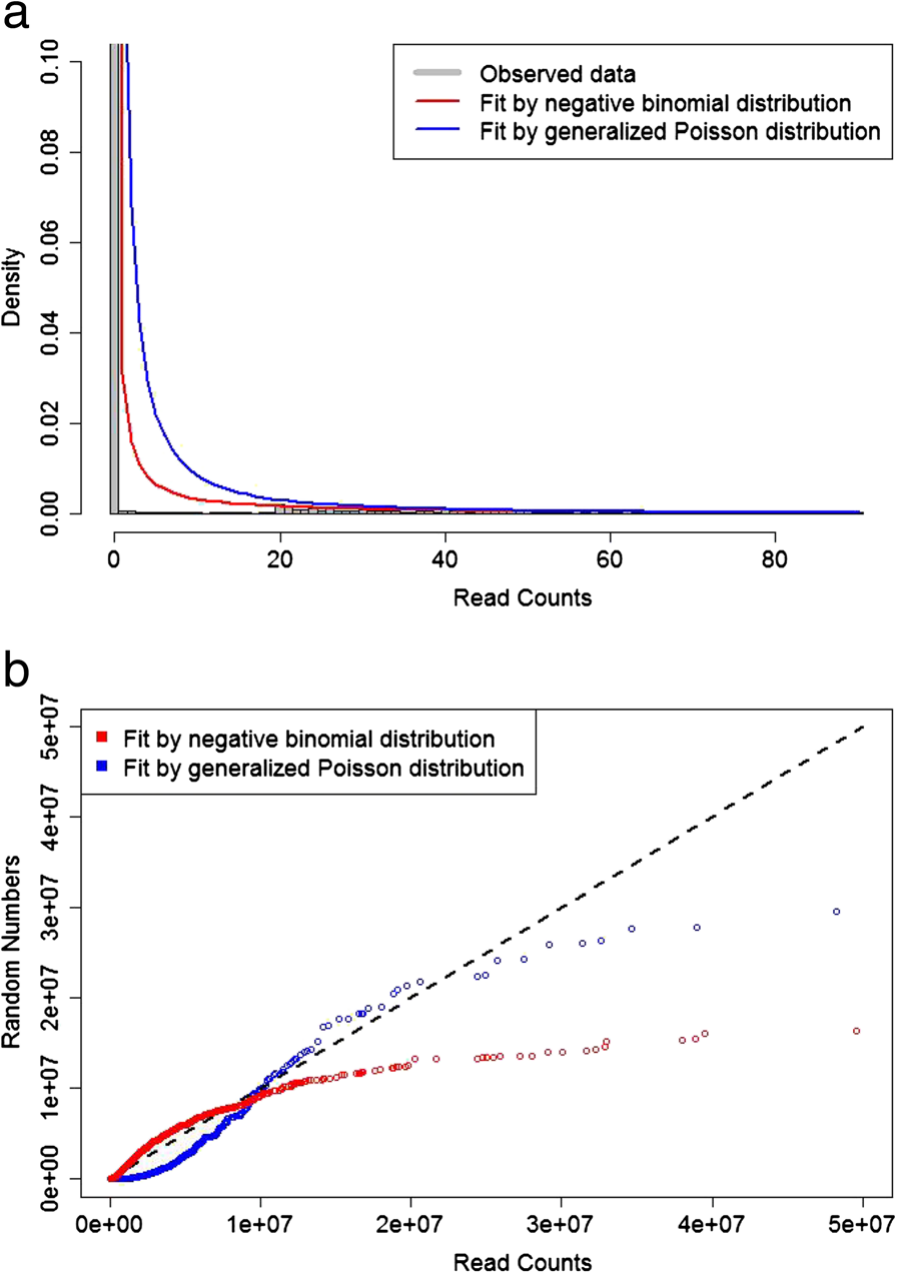
Modeling sequence read counts from RNA-Seq with the NB and GP distributions. **a** Read counts fitted by the NB and GP distributions and **b** QQ plots

To identify biologically important changes in RNA expression, we propose a more accurate and sensitive two-step method for analyzing sequence count data from RNA-Seq experiments (Fig. 1). Here, we implement our method in an R statistical package, termed “deGPS” (https://github.com/LL-LAB-MCW). To speed up permutation tests, deGPS also provides efficient parallel computation using multi-core processors. In Step 1, two different methods based on the GP distribution, namely GP-Quantile and GP-Theta, were developed for normalizing sequence count data. These two GP-based methods differ in parameter estimation and data transformation. Generally, GP distributions fit sequence count data better than NB distributions on transcripts over a wide range of relative abundance in RNA-Seq experiments (Fig. 2). Other commonly used normalization methods including global, quantile [17], locally weighted least squares (Lowess) [18], and trimmed mean method (TMM) [19] for high-throughput data, as is used for microarrays, can be also adopted in deGPS. The latter normalization methods are based on either linear scaling or sample quantiles instead of modeling sequence count data. Normalization in Step 1 removes potential technical artifacts arising from unintended noise, while maintaining the true differences between biological samples.

After data normalization, DE detections are performed in Step 2. We employ the empirical distribution of T-statistics to determine the p-values of DE tests. To obtain empirical distributions, we first randomly shuffle the samples between groups, then calculate T-statistics in permuted samples, and finally merge T-statistics from all transcripts without any averaging as one whole empirical distribution. The number of transcripts analyzed in a typical RNA-Seq experiment is often large, ranging from hundreds to ten of thousands. Using this sampling strategy, reliable empirical distributions can be obtained in small sample sizes. The permutation-based DE test in Step 2 is robust and powerful when sample size is small.

### Simulation strategies

 To evaluate the performance of deGPS, we conducted comprehensive simulations under a range of scenarios comparable to recent RNA-Seq studies. The advantages and disadvantages of each tool are difficult to elicit for a particular small data set. Therefore, we first simulated sequence count data from two large-scale RNA-Seq studies from The Cancer Genome Atlas (TCGA), including 491 miR-Seq libraries (Additional file 1) and 100 mRNA-Seq libraries in human lung tumor tissues (Additional file 2).

To estimate type I error under null hypothesis, we randomly sampled the same number of subjects from our downloaded RNA-Seq datasets into two groups each with 5 subjects. Type I error is defined as the proportion of transcripts with nominal p-values less than 0.05 from statistical tests under null hypothesis. To estimate FDR and true positive rate (TPR) (i.e., statistical power) under alternative hypotheses, we first randomly generated two groups of samples and randomly chose a subset of transcripts. Subsequently, we made two types of changes in the selected transcripts to create DE between two groups. In the “shift” transformation, we added varied quantities (with variations as one fifth of the added values) of read counts into the selected transcripts in either group. In the “scaling” and “shift” transformation, we multiplied the read counts of selected transcripts by varied quantities (with variations as one fifth of the multiplied values) after applying the “shift” transformation (Additional file 3). In our deGPS method, nominal p-values were adjusted by the Benjamini-Hochberg procedure [20]. FDR is defined as the proportion of transcripts identified by a statistical test with a significance level of 0.05 (i.e., adjusted p-values < 0.05) that are indeed false discoveries (i.e., non-DE transcripts); TRP is defined as the proportion of DE transcripts identified by a statistical test with a significance level of 0.05. Each simulation was replicated 1,000 times.

In the real data-driven simulations, sequence count data were normalized by GP-Theta or GP-Quantile methods before applying our permutation-based DE tests. For the purpose of comparison, we also included in the simulation four other normalization methods (namely, Global, Lowess, Quantile, and TMM) that are not based on the GP distribution, but are commonly used for high throughput data such as those from microarray [21,22]. Our DE tests were then applied to the normalized data generated by all of these methods (Additional file 4). We also chose four additional tools, edgeR (v3.6.7), DESeq (v1.16.0), DESeq2 (v1.4.5), and SAMseq (v2.0) which are currently among the top performers of differential analysis of sequence count data [16]. Prior to DE tests, edgeR performs TMM, relative log expression (RLE) or upper quartile for data normalization in its own R package [19]. DESeq and its variant (DESeq2) use a similar RLE approach for data normalization by creating a virtual library that every sample is compared against [5]. Similarly, nominal p-values output from these R packages were adjusted by the Benjamini-Hochberg (BH) procedure for evaluating FDR and TPR [20]. Note that edgeR has multiple user-defined parameter settings while both DESeq and DESeq2 were applied by default setting. We present the results from the most commonly used TMM normalization with glmLRT (named edgeR1) and glmQLF tests (named edgeR2), which generally have better performance than the other setting (Additional file 3). SAMseq were implemented with default parameter setting.

In addition to the above data-driven simulation strategy, we also used compcodeR for benchmarking of DE analysis methods [23]. The compcodeR package provides functionality for simulating realistic RNA-seq count data sets and an interface for implementing several commonly used statistical methods such as DESeq and edgeR for DE analysis. We set the proportion of upregulated transcripts as 50 %, set sample size as 5, 8, and 10 subjects per group, and introduced 0, 0.5, 1.0 and 2.0 % probability of random outliers to model abnormally high counts in RNA-Seq studies. All of the other parameters are default. compcodeR-based simulations were replicated 100 times in each scenario. Type I error, FDR, TPR and AUC were evaluated and compared by its own functions in the compcodeR. It is worth noting that compcodeR simulates sequence count data from NB distributions, which potentially favors DESeq and edgeR.

To evaluate different FDR adjustment methods, we introduced R package fdrtool [24] to further compare BH method [20] to area-based FDR (QVAL) and density-based FDR (LFDR) in compcodeR-based simulations. We took permutation T-statistics instead of p-values in deGPS as the input of fdrtool and extract the QVAL and LFDR from the output. Since sample sizes in RNA-Seq experiments are typically small, the estimated variances and their associated T-statistics used in permutation tests are probably highly variable. We thus compared ordinary T-statistic to regularized T statistic in permutation tests for DE detection. Regularized T-statistic was implemented in R package st [25].

### Type I errors and false positive rates

**Fig. 3:**
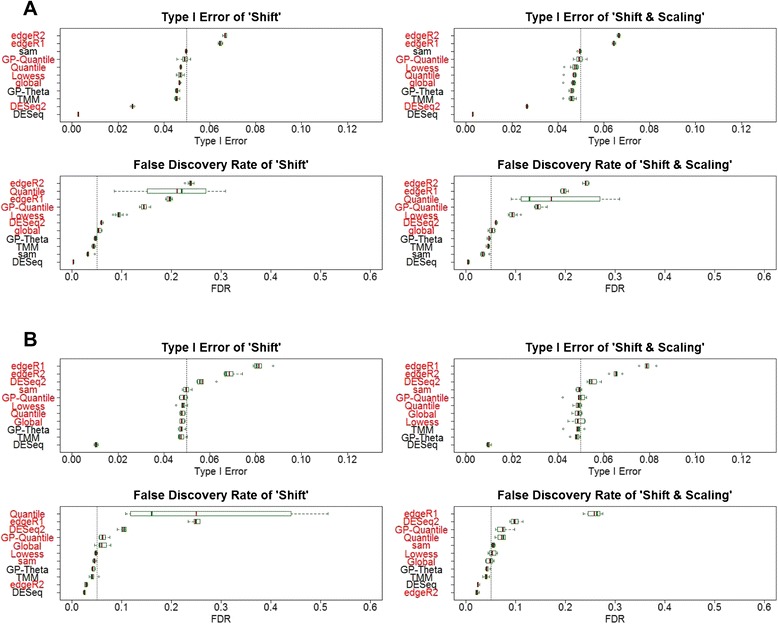
Type I error and false discovery rate. Data were simulated from large-scale TCGA lung cancer sequencing studies, **a** miRNA and **b** mRNA. Two different types of data transformation, “shift” and “scaling & shift” were applied. Boxplots summarize type I error and false discovery rate of different statistical methods for DE detection under a wide range of simulations. Methods in red font are those do not have correct type I error and/or false discovery rate

We first evaluated type I error and FDR of different methods in datasets simulated from two large-scale RNA-Seq studies, including 491 miRNA and 100 mRNA TCGA samples (Fig. 3). FDR is used for quantifying the rate of false discoveries when multiple hypothesis testing is concerned especially in RNA-Seq experiments. Among these methods, only three methods (GP-Theta, TMM and DESeq) can precisely control both type I error and FDR in both miRNA and mRNA datasets. SAMseq has correct type I error and FDR in miR-Seq dataset, but inflates type I error and FDR in mRNA-Seq dataset. DESeq is the most conservative among these methods in terms of type I error and FDR. Its variant DESeq2 becomes less conservative but leads to higher FDR than expected. edgeR appears to be unable to control both type I error and FDR in all scenarios (Fig. 3 and Additional file 5).

Six of these methods (GP-Theta, GP-Quantile, Global, Lowess, Quantile, and TMM) use different strategies of data normalization, but use the same DE tests as deGPS. They yield very different type I error and FDR. Only GP-Theta and TMM are able to control both type I error and FDR at the desired level; whereas the other four methods have inflated type I error and/or FDR. These results suggest data normalization has substantial impacts on the performance of DE tests in terms of type I error and FDR.

### True positive rates

**Fig. 4:**
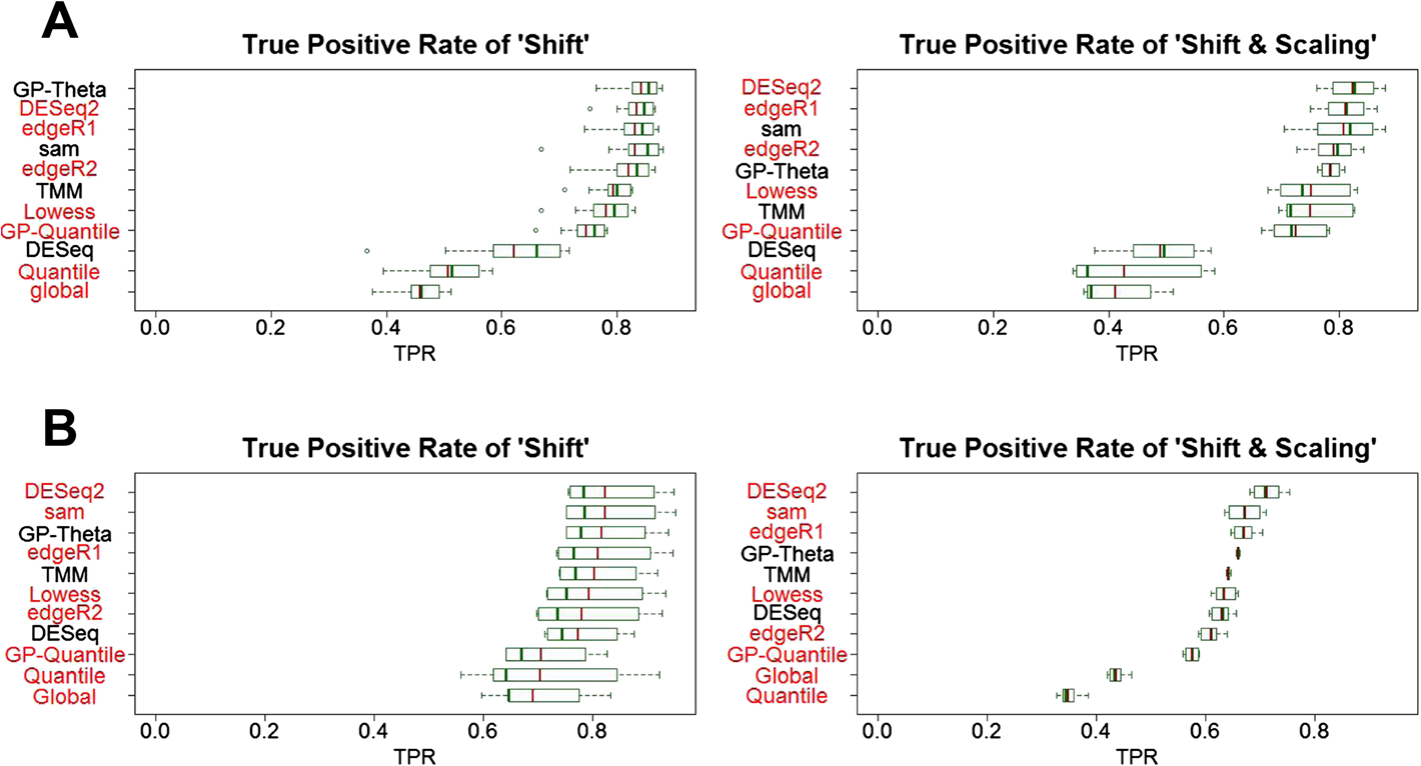
True positive rate. **a** miRNA and (**b**) mRNA. True positive rate (TPR) can be interpreted as statistical power

Next, we evaluated TPR (i.e., statistical power) of different methods in these RNA-Seq datasets (Fig. 4 and Additional file 6). These methods show different TPR among different RNA-Seq datasets. GP-Theta consistently produces the highest TPR among the methods that also have correct type I error and FDR in both miRNA and mRNA datasets. SAMseq has roughly similar TPR to deGPS without regard to the consequence of type I error and FDR. DESeq2 has improved TPR, but at the cost of inflated type I error and FDR, when compared with its original version DESeq. Generally, edgeR has high TPR but also exhibits high FDR too.

We also observed that data normalization dramatically influences statistical power of DE tests. Although the same DE tests were applied after data normalization, six different normalization methods result in varied TPR. Besides GP-Theta, TMM performs reasonably better than the other four methods.

### Sensitivity and specificity

**Fig. 5:**
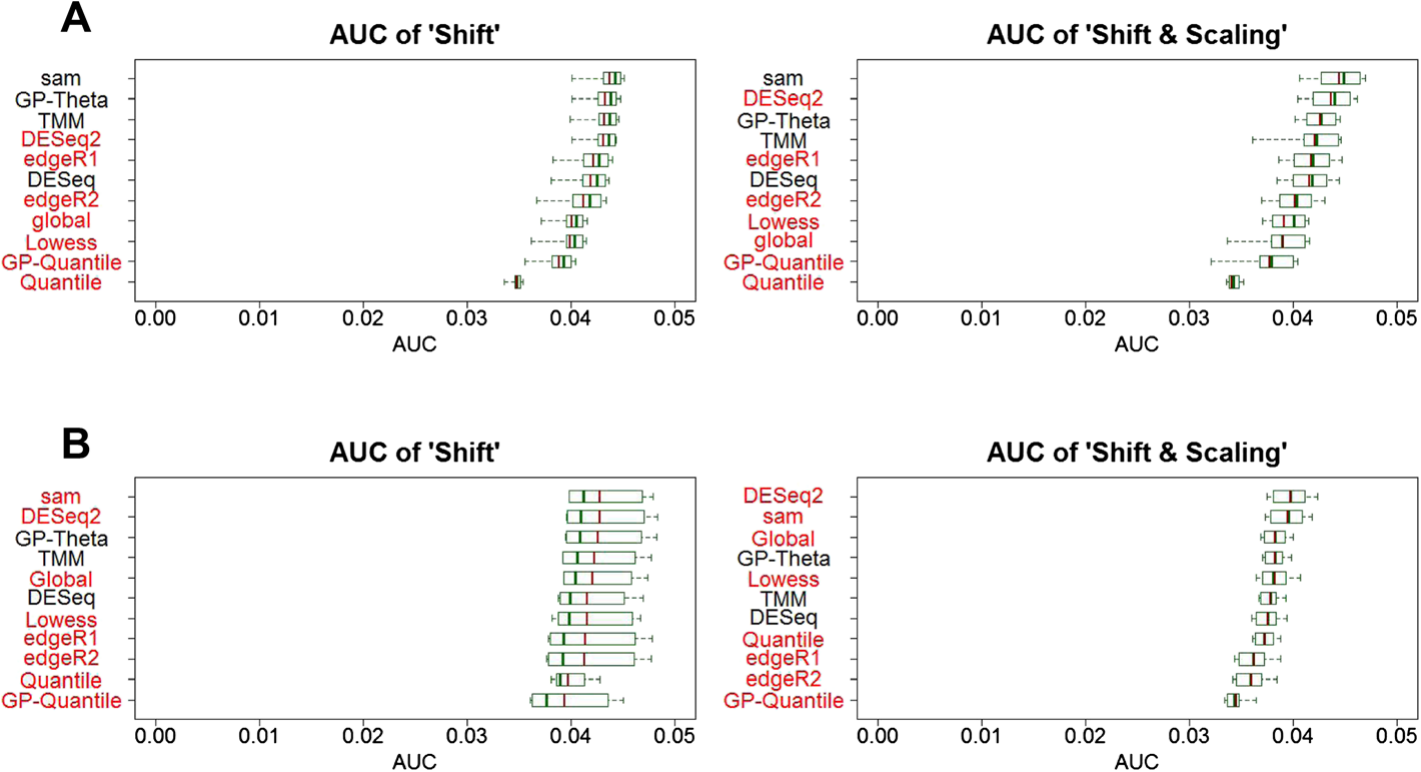
Sensitivity and specificity. **a** miRNA and **b** mRNA. The AUC with false positive rate less than 0.05 was calculated. Boxplots summarize AUC values from a wide range of simulation settings. TPR, true positive rate; FPR, false positive rate

We compared deGPS with other methods in terms of sensitivity and specificity in these two RNA-Seq studies. We thus calculated the receiver operating characteristic (ROC) curve and area under curve (AUC) of different methods to measure their sensitivity and specificity (Fig. 5 and Additional files 7). For the clearer presentation, AUC with false positive rate (FPR) less than 0.05 was calculated. In general, SAMseq, GP-Theta, DESeq2 and TMM are the top four performers for DE analysis of sequence count data according to the AUC metric. Among the methods that have correct type I error and FDR, GP-Theta performs the best as it has the largest AUC. DESeq2 often has higher AUC than its original version DESeq. Generally, DESeq and its variant DESeq2 perform better than edgeR in mRNA datasets in terms of AUC, whereas their performances are comparable in miRNA datasets. The normalization methods other than GP-Theta and TMM usually result in lower AUC.

### Benchmark data

**Fig. 6:**
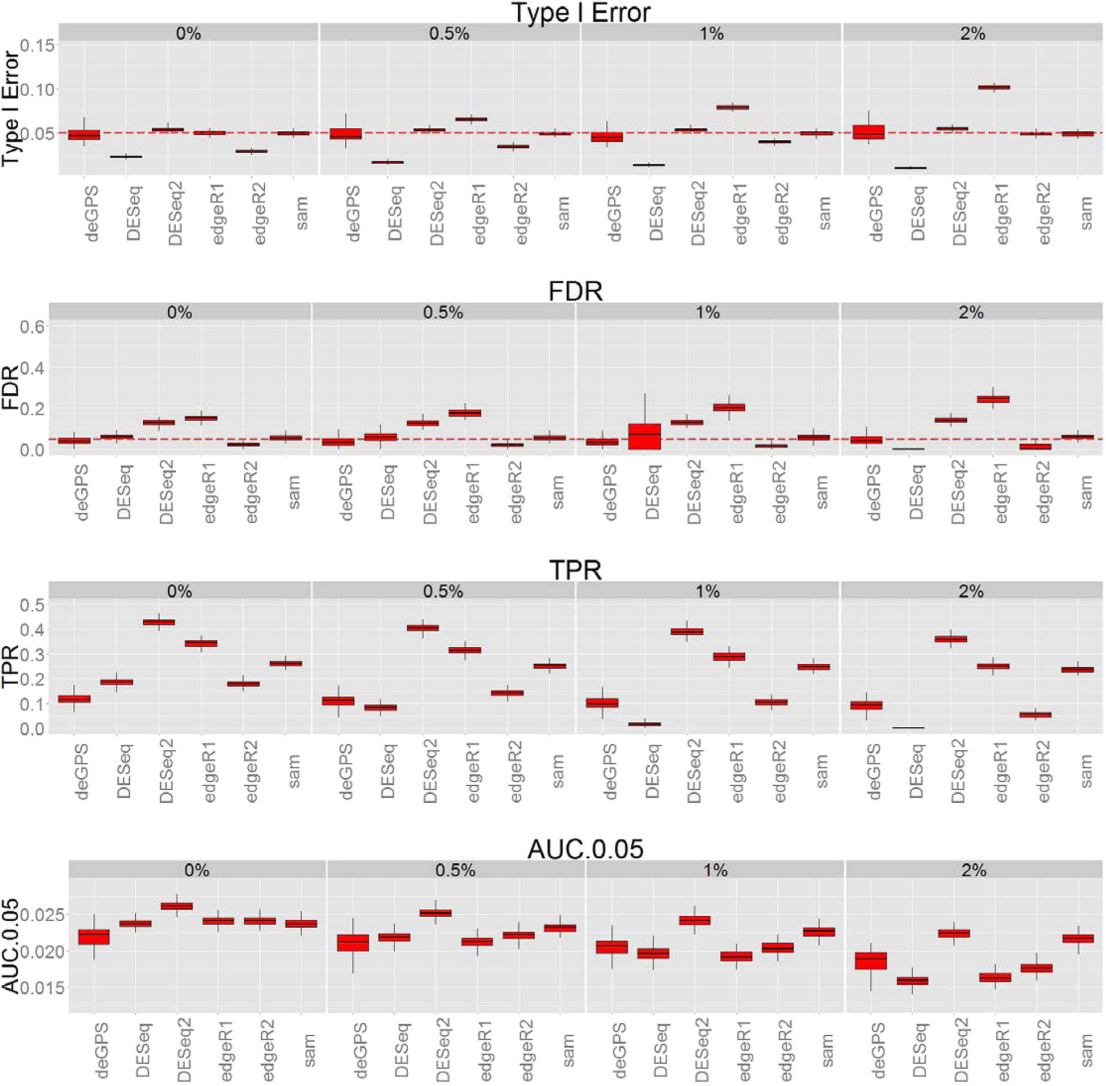
Benchmark data from compcodeR. Type I error rate, FDR, TPR and AUC are evaluated under 0, 0.5, 1 and 2 % of outliers in RNA-Seq data. Sample size is 5 subjects per group

We further compared deGPS with SAMseq, DESeq and edgeR using compcodeR. compcodeR is an R package for benchmarking of DE analysis methods, in particular methods developed for analyzing RNA-Seq data [23]. In the analysis, deGPS with GP-Theta normalization, SAMSeq, DESeq and DESeq2, and edgeR1 and edgeR2 were evaluated in benchmark data (Fig. 6 and Additional file 8).

In compcodeR-based simulations, both deGPS and SAMSeq consistently control both type I error and FDR and are robust against the occurrence of random outliers in RNA-Seq experiments; whereas DESeq2 and edgeR1 are not able to control type I error and/or FDR in most of scenarios. DESeq is still conservative in terms of type I error, but its ability of FDR control varies among different levels of random outliers and samples. edgeR2 generally performs much better than edgeR1 in terms of FDR control in compcodeR-based simulations. edgeR1 is based on generalized linear model in which regular likelihood ratio test (LRT) is performed; whereas edgeR2 replaces the Chi-square approximation to the LRT statistic with a quasi-likelihood F-test [26].

In terms of TPR and AUC, SAMseq performs slightly better than deGPS, but the difference between these two methods becomes small when increasing sample sizes from 5 to 8 subjects per group (Fig. 6 and Additional file 8). edgeR2 performs similarly to deGPS in RNA-Seq data without random outliers or very low proportion of outliers (i.e., <0.5 %) . However, deGPS outperforms edgeR2 when random outliers increase up to 1 % in RNA-Seq data. Interestingly, deGPS achieves similar TPR under different levels of random outliers, suggesting it is a robust approach for DE analysis in the presence of abnormal high sequence read counts in particular transcripts in RNA-Seq experiments. It should be also noted that both DESeq and edgeR model sequence count data with NB distribution; whereas deGPS is based on GP distribution. Therefore, compcodeR benchmark analysis that simulates sequence count data from NB distributions may favor DESeq and edgeR and thus overestimate their performance as compared with deGPS in real RNA-Seq data.

We also evaluated effects of different FDR adjustment methods on the performance of deGPS. The median FDR of QVAL or LFDR is a little smaller than that of BH method although the later can precisely control both FDR and type I error. QVAL and LFDR do not always outperform BH method when repeating simulation in each scenario as they have much bigger interquartile range in the boxplot (Additional file 9). It may worth further investigation why the performances of these three FDR adjusting methods differ from case to case. Finally, we compared ordinary T-statistic with regularized T-statistic in permutation tests for DE detection. The simulation results showed that, based on deGPS-transformed data, ordinary T-statistic has a little higher TPR and is generally comparable with regularized T-statistic in terms of type I error and AUC (Additional file 9).

### Real data analysis of the developmental transcriptome of Drosophila

**Fig. 7:**
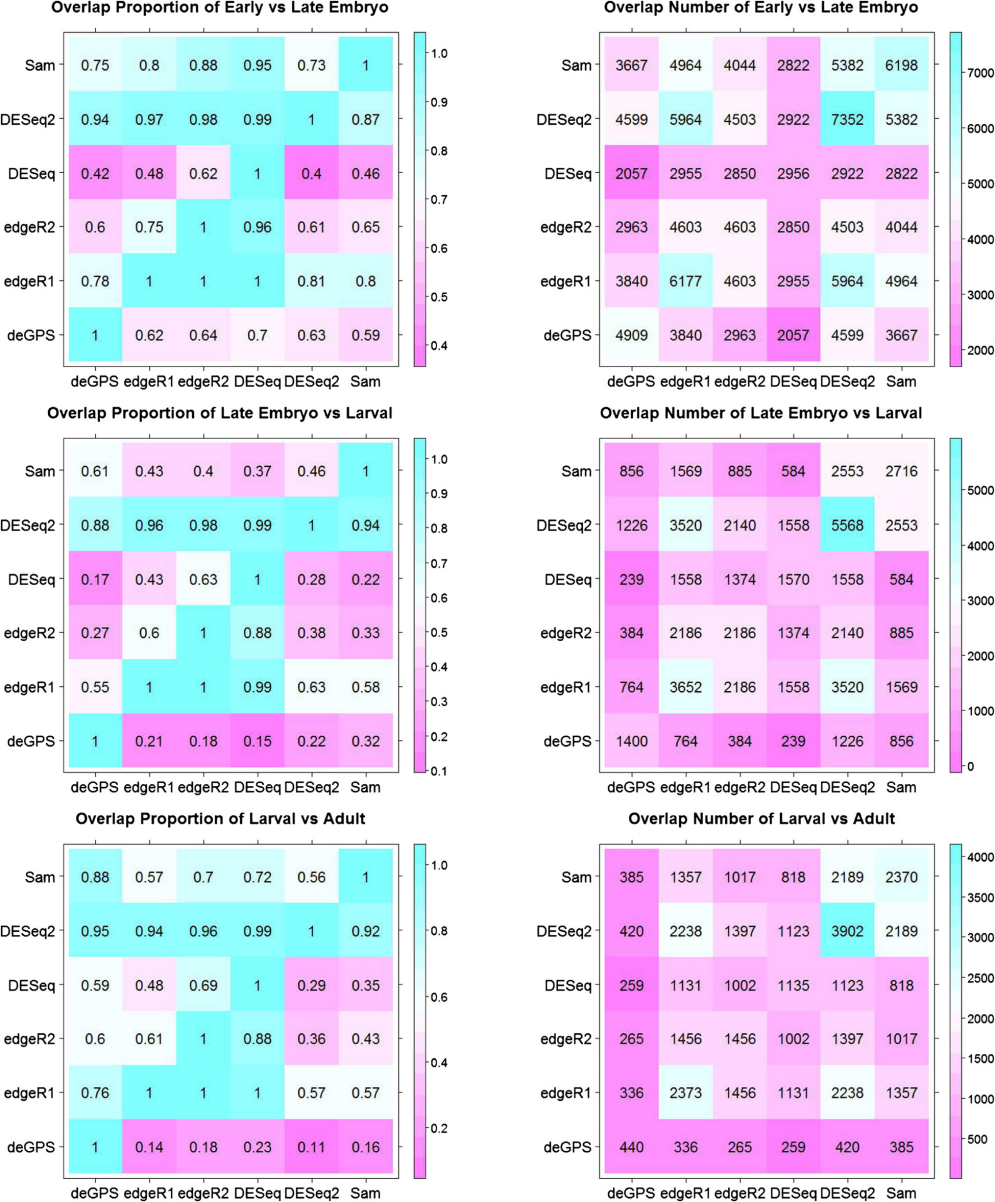
Analysis of the development transcriptome of Drosophila Melanogaster. Four development stages (early embryo, later embryo, larval and adult) were analyzed (Graveley, et al., 2011). The numbers of genes differentially expressed between two adjacent stages are presented at a FDR threshold of 0.05. The “overlap proportion” is calculated as dividing overlap numbers by its column’s DEs

In addition to simulated datasets, we also analyzed the developmental transcriptome of Drosophila melanogaster (Fig. 7 and Additional file 10) [27]. We compared six different methods (i.e., deGPS, SAMseq, DESeq, DESeq2, edgeR1 and edgeR2) to identify genes that were differentially expressed between four development stages of Drosophila, which include early embryo (0 to 12 days), late embryo (13 to 24 days), larval and adult stages. Each stage contains 6 RNA-Seq samples. The RNA-Seq read count data in 14,869 genes from these 24 samples were downloaded from http://bowtie-bio.sourceforge.net/recount [28]. Prior to the analysis, we filtered genes without any read counts in all samples from any two compared groups. Similar to the above simulations, the BH procedure was used to control FDR [20], and all genes found to be DE at a FDR threshold of 0.05 were considered significantly DE. As expected, there were a large number of developmental-regulated DE genes in early embryo development, compared with adult Drosophila. Generally, edgeR1 and DESeq2 identified the largest number of DE genes than the other methods. This is perhaps due to their failure in controlling FDR, as observed in simulations. DESeq is the most conservative and identified the smallest number of DE genes among these methods. edgeR methods show extremely high concordance; all of DE genes that were identified by edgeR2 were identified by edgeR1. Similar observations are also true in DESeq methods; about 99 % of DE genes that were identified by DESeq were identified by DESeq2. Approximately 70, 70 and 87 % of DE genes found by deGPS overlap with SAMseq, edgeR1 and DESeq2, respectively.

Next, we evaluated the ability of the above methods to control type I error and false positive numbers. We randomly assigned equal number of subjects (without replacement) from the same development stages into two groups of 5 subjects each. Each group contained equal number of subjects from the same development stages and thus had similar gene expression profiles. Therefore, we expected that no genes are truly DE when comparing these two synthetic groups. Nevertheless, among 100 simulations, these methods identified DE genes ranging from 16 to 277 false positives per genome scan. deGPS found the lowest number of false positives, whereas edgeR1 found the highest number of false positives. edgeR1 inflates type I error, whereas the other four methods can control type I error at the desired level (Additional file 11).

## Discussion

In this study, we developed a novel tool, deGPS, for data normalization and DE detection in RNA-Seq studies. deGPS shows improved results for analyzing count-based expression data in most cases through comprehensive simulations. Among 11 methods evaluated in our simulations, it is the only one that can precisely control type I error and FDR in all of scenarios while maintaining high statistical power for DE detection. Good performance of deGPS results from two significant methodological improvements. First, the newly proposed normalization methods model sequence count data by using GP distribution. Data normalization has substantial impact on the performance of statistical methods for DE analysis of sequence count data. Among the six normalization methods evaluated in our study, GP-Theta achieved highest power and AUC while controlling type I error and FDR in either real data-driven simulations or compcodeR-based benchmark data. One possible reason why GP-Theta outperforms the other normalization methods is that it gives a definite estimate of how much the sample mean should be shrunk to alleviate the impact of overdispersion. Second, the regular permutation-based DE tests in deGPS are robust and powerful. Though the data may be skewed, simulations have proved that it is appropriate to pull T-statistics from all transcripts to form one whole empirical distribution. Using this strategy, reliable empirical distributions can be obtained in small sample sizes where many statistical models are prone to inflated type I error and/or FDR. Appropriate use of FDR adjustment methods and regularized T-statistics in permutations may further improve the performance of deGPS. This requires further investigation in future studies.

We compared our deGPS with edgeR, DESeq and SAMseq, which are currently among the top performers for DE analysis of sequence count data [16]. There are methodological distinctions between deGPS and edgeR/DESeq. Our deGPS assumes a GP distribution on the data for a single library across all genes, whereas edgeR and DESeq assumes a NB distribution on the data for a single gene across non-differenentially expressed libraries. Our simulations showed that DESeq is relatively conservative in terms of type I error and is prone to inflated FDR when outliers are introduced to RNA-Seq data. Its variant DESeq2 becomes less conservative and has an increased power but at the cost of poor FDR control. edgeR1 appeared unable to control type I error and FDR in either real data-driven simulations or compcodeR-based benchmark data. edgeR1 method uses LRT statistics that are approximated by a Chi-square distribution, whereas edgeR2 replaces the Chi-square approximation to the LRT statistic with a quasi-likelihood F-test [26]. As a result, edgeR2 has improved FDR control as compared with edgeR1 in most cases. SAMseq is a nonparametric method for finding DE. It performs reasonably better in compcodeR-based simulations, whereas it inflates type I error and FDR in real data-driven simulations from mRNA datasets.

It is not uncommon that some extremely high abundant transcripts (e.g., pseudogenes, ribosomal RNAs, mitochondrial RNAs, contaminant mRNAs and unannotated RNAs) are presented in RNA-Seq data, for example, as seen in the above Drosophila RNA-Seq data (Additional file 12) [27]. These abnormally high read counts (i.e., outliers) in RNA-Seq data will lead to increased numbers of falsely declared DE genes if standard normalization is applied. For example, DESeq, DESeq2 and edgeR1 inflate FDR and lose TPR (i.e., power) when increasing the proportion of outliers up to 0.5 % in RNA-Seq data. Although edgeR2 maintains correct FDR, TPR is dramatically decreased with the increase of outliers in the data. Interestingly, our deGPS consistently controls both type I error and FDR, and maintains similar TPR under different levels of random outliers. This suggests that deGPS is a robust approach for DE analysis in the presence of abnormal high sequence read counts in RNA-Seq samples. In the GP-Theta method, normalization factor is estimated as sample mean multiplied by $\left(1-\widehat{\lambda}\right)$ where $\widehat{\lambda}$ is an overdispersion parameter accounting for unexpectedly high variability in sequence count data. We observed that large variability of $1/\left(1-\widehat{\lambda}\right)$ exists across RNA-Seq samples from the analysis of two large-scale TCGA data (Additional file 13), suggesting the necessity of shrinkage normalization strategy in these overdispersed count data. Such shrinkage strategy in the analysis helps maintain statistical power and robustness of DE detection.

There are several limitations in deGPS. First, the permutation traversing all the probabilities becomes computationally time-consuming when the sample size increases, though a maximum of permutations can be specified to avoid the problem. To partially alleviate the computational burden, deGPS provides efficient parallel computation in multi-core processors to speed up permutation tests. Runtime of deGPS for RNA-Seq experiments with less than 10 subjects per group is comparable, if parallel computation is applied, to edgeR and DESeq which are currently one of the fastest and most commonly used R packages for DE analysis of RNA-Seq data (Additional file 14). For example, deGPS takes about 3 min for analyzing the Drosophila developmental transcriptome on a Dell PowerEdge r620 with Intel Xeon E5-2660 2.20 Ghz dual-socket 8-core. Although sample sizes will affect runtime of deGPS, it is worth noting that as compared with other methods, permutation-based DE detection implemented in deGPS is robust against different sample sizes. Second, deGPS cannot handle complex experimental designs. Only two-group differential test is currently considered in deGPS. However, our GP-Theta normalization method can be potentially adopted in complex design of RNA-seq experiments or using other statistics instead of a t statistic. Third, it may be inappropriate to compare two groups with library sizes of all samples in one group several times consistently larger than another. Under such very rare circumstances, the shrinkage on sample mean is heavy because of the severely overdispersed read counts. As a result, the normalization factors may not increase as fast as the library size does. The variations within groups may therefore not be large enough to eliminate the large library size differences so that empirical distribution of t statistics may be biased. In that case, TMM normalization is suggested in the application of deGPS package. Fourth, in mRNA data, our method is currently applicable to gene-level read count data while the application on position-level read count data remains further investigations.

In summary, we developed a powerful and robust tool for differential analysis of count-based expression of RNA-Seq data. We implemented our methods in an R package deGPS with parallel computations. deGPS performs better than existing methods in most cases. It is a robust approach against the occurrence of data outliers in RNA-Seq experiments. Beyond RNA-Seq, deGPS has the potential to significantly enhance future data analysis efforts from many other high-throughput platforms such as ChIP-Seq, MBD-Seq and RIP-Seq [29].

## Methods

### GP distribution

Sequence count data, *X*, observed in a RNA-Seq experiment can be modeled with a GP distribution with parameters θ and λ:

1 $$ \Pr \left(\mathrm{X}=\mathrm{x}\right)=\left\{\begin{array}{lll}\hfill & \frac{\uptheta {\left(\uptheta +\mathrm{x}\uplambda \right)}^{\mathrm{x}\hbox{-} 1}{{\mathrm{e}}^{\hbox{-} \uptheta \hbox{-} \mathrm{x}}}^{\uplambda}}{\mathrm{x}!},\hfill & \mathrm{x}=0,1,2,\dots \hfill \\ {}0\hfill & \hfill & \mathrm{f}\mathrm{o}\mathrm{r}\;\mathrm{x}>q\; if\;\lambda <0\hfill \end{array}\right. $$

where θ > 0, $\max \left(-1,-\frac{\uptheta}{\mathrm{q}}\right)\le \uplambda \le 1,$, and q(≥4) is the largest positive integer for which θ + qλ > 0 when λ < 0. The mean of *X* is θ(1 − λ)^− 1^ and the variance of *X* is θ(1 − λ)^− 3^. When λ = 0, GP becomes a Poisson. The parameter θ is the mean for the natural Poisson process. The parameter λ is the average rate of effort that the subjects are making to deviate from the process. A positive value of λ indicates that the subjects are making an effort to accelerate the natural process while the negative one denotes an effort to retard the process [30]. In the context of RNA-Seq, θ represents the average number of reads mapped to transcripts in a sample. It is correlated to the depth of sequence coverage and total reads mapped to reference genome in the sample. λ represents the bias during the sample preparation and sequencing process [31]. Note that all the fitted λs are always far away from zero, which suggests sequence count data is highly over-dispersed in RNA experiments. It is worth noting that deGPS models gene- or transcript-level sequence count data within the same sample. This is distinct from GPseq that instead models position-level count data [31].

The maximum likelihood estimate (MLE) of λ in the GP model (1) can be obtained by solving the following equation:

2 $$ {\displaystyle {\sum}_{\mathrm{i}=1}^{\mathrm{n}}\frac{{\mathrm{X}}_{\mathrm{i}}\left(1-{\mathrm{X}}_{\mathrm{i}}\right)}{\overline{\mathrm{X}}+\left({\mathrm{X}}_{\mathrm{i}}-\overline{\mathrm{X}}\right)\uplambda}}-\mathrm{n}\overline{\mathrm{X}}=0 $$

where $\overline{\mathrm{X}}={\displaystyle {\sum}_{\mathrm{i}=1}^{\mathrm{n}}{\mathrm{X}}_{\mathrm{i}}/\mathrm{n}}$ and is the sample mean of reads mapped to transcripts. The MLE of θ can be estimated as $\overline{\mathrm{X}}\left(1-\widehat{\uplambda}\right)$.

### Normalization methods

We propose two new normalization methods for sequence count data based on the above GP distribution: GP-Quantile and GP-Theta. The GP-Quantile method fits every sample in the data with GP distribution, and maps every read count to the corresponding probability, P(X < x), of the fitted GP. Despite that read counts in every sample are normalized between 0 and 1, the data information may be lost during the GP-Quantile normalization process.

In the GP-Theta method, read counts from each sample are divided by the parameter θ of the fitted GP distribution. The MLE of θ is $\overline{\mathrm{X}}\left(1-\widehat{\uplambda}\right)$ where $\widehat{\uplambda}$ is the MLE of the over-dispersion parameter λ and $\overline{\mathrm{X}}$ is the sample mean of sequence reads mapped to transcripts. This MLE $\widehat{\uptheta}$ can be treated as a shrunk value of $\overline{\mathrm{X}}$. A major purpose of the GP-Theta method is to remove sample bias due to depth of sequence coverage in RNA-Seq experiments. Similar ideas were previously used for the normalization of RNA-Seq data such as trimmed mean method (TMM) [19].

### Differential expression tests

After the data normalization, a procedure using empirical distributional of *T*-test statistic is conducted in our DE test. To eliminate potential technical noise arising from RNA-Seq experiments, *T*-test statistics are calculated after normalizations:

$$ \mathrm{T}.\mathrm{stat}\left(\mathrm{X}\hbox{'},\mathrm{Y}\hbox{'}\right)=\frac{\mathrm{Mean}\left(\mathrm{X}\hbox{'}\right)\hbox{-} \mathrm{Mean}\left(\mathrm{Y}\hbox{'}\right)}{\sqrt{\mathrm{Var}\left(\mathrm{X}\hbox{'}\right)/{\mathrm{N}}_{\mathrm{x}\hbox{'}}+\mathrm{V}\mathrm{a}\mathrm{r}\left(\mathrm{Y}\hbox{'}\right)/{\mathrm{N}}_{\mathrm{y}\hbox{'}}}} $$

where “Var” is the variance function and “Mean” is the mean value of read count of a transcript in the sample. X’ and Y’ are GP-transformed read counts from two groups of samples; N_x’_ and N_y’_ are sample sizes of the two groups.

We propose to use empirical distribution of T-statistics to determine the p-values of DE tests. We generate empirical distributions by randomly shuffling the samples into two groups and calculate *T*-test statistics for each transcript in the permutated samples. Due to the abundance of the transcripts, our permutation strategy can produce reliable empirical distributions even with small sample sizes (e.g., two samples for each group) that are still common in RNA-Seq experiments. The p-values are therefore calculated according to the empirical distribution of T statistics. However, the pooled t-statistics is mixture of a “null group” of statistics corresponding to non DE genes and an “alternative” group corresponding to DE genes. Thus we also include fdrtool [24] to adjust p values in our R package.

The estimated variances and thus T-statistics used in permutation tests are probably highly variable due to a typically small sample sizes in RNA-Seq experiments. Instead of the above ordinary T-statistics, regularized T-statistics, implemented in R package st, are also included in our deGPS.

The real data analysis of the developmental transcriptome of Drosophila can be found in our released R package–deGPS (https://github.com/LL-LAB-MCW). compcodeR-based simulations can be repeated by the R codes which are available in Additional file 15.

### Availability of supporting data

The data sets supporting the results of this article are included within the article and its additional files.

## Additional files

Additional file 1: Table S1. -TCGA samples used in microRNA-Seq simulations. Additional file 2: Table S2. -TCGA samples used in mRNA-Seq simulations. Additional file 3: Document S1. -Simulation settings. Additional file 4: Figure S1. -A flowchart for method comparisons in simulations. Additional file 5: Figure S2. -Type I error and false discovery rate of edgeR with different parameter settings. Different parameter settings in edgeR were defined in Document S1. Methods in red font are those inflate type I error and/or false discovery rate. Additional file 6: Figure S3. -True positive rate of edgeR with different parameter settings. Methods in red font are those inflate type I error and/or false discovery rate. Additional file 7: Figure S4. -AUC of edgeR with different parameter settings. Methods in red font are those inflate type I error and/or false discovery rate. Additional file 8: Figure S5. -Simulation results from compcodeR. Sample size was set as 8 subjects per group. Note that edgeR2 does not control correct type I error when increasing sample size although its FDR is at the desired level. Additional file 9: Figure S6. -Effects of different FDR adjustment methods and T-statistics on the performance on deGPS. Sample sizes were set as (A) 5 and (B) 8 subjects per group. The default FDR adjustment of deGPS is BH method; two additional FDR adjustments QVAL and LFDR were evaluated. The default statistic of deGPS is ordinary T-statistic in permutations; regularized T-statistic (st) was evaluated. Additional file 10: Figure S7. -Genes differentially expressed between any two non-adjacent developmental stages of Drosophila melanogaster Additional file 11: Figure S8. -Average false positive number and type I error when comparing two groups that are randomly and equally sampled from different developmental stages of Drosophila melanogaster. Additional file 12: Figure S9. -Outliers in Drosophila data. Blue points represent the logarithm of the difference between quantiles and five times sample mean. And red points represent the logarithm of the difference between quantiles and ten times sample mean. compcodeR generates outliers by multiplying the read counts by 5–10. The figure shows that top 2 % read counts are larger than 5 times sample mean and top 1 % read counts are larger than 10 times sample mean. Though sample mean may not represent the read counts randomly generated by compcodeR properly, we can conclude that up to 1-2 % random outliers are not rare in real data. Additional file 13: Figure S10. -Overdispersion of sequence count data in RNA-Seq. (A) Histogram of 1/(1-λ ^) (in logarithm scale), and (B) Sample variance is far away from its mean. Sequence count data were fitted with GP distribution for each sample from TCGA. 1/(1-λ ^) measures the extent of the departure of the data from Poisson distribution. Additional file 14: Table S3. -Running times of different R packages for analyzing the development transcriptome of Drosophila. Additional file 15: R codes-Repeat compcodeR-based simulations.

## Abbreviations

AUC: Area under curve
ChIP-Seq: Chromatin immunoprecipitation sequencing
DE: Differential expression
FDR: False discovery rate
FPR: False positive rate
GP: Generalized Poisson
lncRNA: Long noncoding RNAs
Lowess: Locally weighted least squares
LRT: Likelihood ratio test
MBD-Seq: Methyl-CpG binding domain protein-enriched genome sequencing
miRNA: MicroRNA
mRNA: Messenger RNA
NB: Negative binomial
NGS: Next-generation sequencing
RIP-seq: RNA-immunoprecipitation sequencing
RNA-Seq: RNA sequencing
ROC: Operating characteristic curve
siRNA: Small interfering RNAs
TCTA: The cancer Genome Atlas
TMM: Trimmed mean method
TPR: True positive rate
