# Supplementary material for: deGPS is a powerful tool for detecting differential expression in RNA-sequencing studies
Source: BMC Genomics. 2015 Jun 13;16(1):455. doi: 10.1186/s12864-015-1676-0 (PMC4465298; doi:10.1186/s12864-015-1676-0)
Supplement: Additional file 1: Table S1. — -TCGA samples used in microRNA-Seq simulations. [file 12864_2015_1676_MOESM1_ESM.pdf]

Table S1 TCGA samples used in microRNA-Seq simulations.

order	sample_id	order	sample_id
1	TCGA-05-4244-01A-01T-1108-13	51	TCGA-44-2656-01A-02T-0947-13
2	TCGA-05-4249-01A-01T-1108-13	52	TCGA-44-2657-01A-01T-1108-13
3	TCGA-05-4250-01A-01T-1108-13	53	TCGA-44-2659-01A-01T-0947-13
4	TCGA-05-4382-01A-01T-1207-13	54	TCGA-44-2661-01A-01T-1108-13
5	TCGA-05-4384-01A-01T-1754-13	55	TCGA-44-2662-01A-01T-0947-13
6	TCGA-05-4389-01A-01T-1207-13	56	TCGA-44-2665-01A-01T-0947-13
7	TCGA-05-4390-01A-02T-1754-13	57	TCGA-44-2666-01A-01T-0947-13
8	TCGA-05-4395-01A-01T-1207-13	58	TCGA-44-2668-01A-01T-0947-13
9	TCGA-05-4396-01A-21H-1857-13	59	TCGA-44-3396-01A-01T-1207-13
10	TCGA-05-4397-01A-01T-1207-13	60	TCGA-44-3398-01A-01T-1108-13
11	TCGA-05-4398-01A-01T-1207-13	61	TCGA-44-3918-01A-01T-1108-13
12	TCGA-05-4402-01A-01T-1207-13	62	TCGA-44-3919-01A-02T-1108-13
13	TCGA-05-4403-01A-01T-1207-13	63	TCGA-44-4112-01A-01T-1108-13
14	TCGA-05-4405-01A-21H-1857-13	64	TCGA-44-5643-01A-01T-1627-13
15	TCGA-05-4410-01A-21H-1857-13	65	TCGA-44-5644-01A-21H-2038-13
16	TCGA-05-4415-01A-22H-1857-13	66	TCGA-44-5645-01A-01T-1627-13
17	TCGA-05-4417-01A-22H-1857-13	67	TCGA-44-6144-01A-11H-1754-13
18	TCGA-05-4418-01A-01T-1207-13	68	TCGA-44-6145-01A-11H-1754-13
19	TCGA-05-4420-01A-01T-1207-13	69	TCGA-44-6146-01A.r3
20	TCGA-05-4422-01A-01T-1207-13	70	TCGA-44-6147-01A.r3
21	TCGA-05-4424-01A-22H-1857-13	71	TCGA-44-6148-01A-11H-1754-13
22	TCGA-05-4425-01A-01T-1754-13	72	TCGA-44-6774-01A-21H-1857-13
23	TCGA-05-4426-01A-01T-1207-13	73	TCGA-44-6775-01A.r3
24	TCGA-05-4427-01A-21H-1857-13	74	TCGA-44-6776-01A-11H-1857-13
25	TCGA-05-4430-01A-02T-1207-13	75	TCGA-44-6777-01A-11H-1857-13
26	TCGA-05-4432-01A-01T-1207-13	76	TCGA-44-6778-01A-11H-1857-13
27	TCGA-05-4433-01A-22H-1857-13	77	TCGA-44-6779-01A-11H-1857-13
28	TCGA-05-4434-01A-01T-1207-13	78	TCGA-44-7659-01A-11H-2065-13
29	TCGA-05-5420-01A-01T-1627-13	79	TCGA-44-7660-01A-11H-2065-13
30	TCGA-05-5423-01A-01T-1627-13	80	TCGA-44-7661-01A-11H-2065-13
31	TCGA-05-5425-01A-02T-1627-13	81	TCGA-44-7662-01A-11H-2065-13
32	TCGA-05-5428-01A-01T-1627-13	82	TCGA-44-7667-01A-31H-2065-13
33	TCGA-05-5429-01A-01T-1627-13	83	TCGA-44-7669-01A-21H-2065-13
34	TCGA-05-5715-01A-01T-1627-13	84	TCGA-44-7670-01A-11H-2065-13
35	TCGA-35-3615-01A-01T-0947-13	85	TCGA-44-7671-01A-11H-2065-13
36	TCGA-35-4122-01A-01T-1108-13	86	TCGA-44-7672-01A-11H-2065-13
37	TCGA-35-4123-01A-01T-1108-13	87	TCGA-44-8117-01A-11H-2240-13
38	TCGA-35-5375-01A-01T-1627-13	88	TCGA-44-8119-01A-11H-2240-13
39	TCGA-38-4625-01A-01T-1207-13	89	TCGA-44-8120-01A-11H-2240-13
40	TCGA-38-4626-01A-01T-1207-13	90	TCGA-44-A479-01A-31H-A24G-13
41	TCGA-38-4627-01A-01T-1207-13	91	TCGA-44-A47A-01A-21H-A24G-13
42	TCGA-38-4628-01A-01T-1207-13	92	TCGA-44-A47B-01A-11H-A24G-13
43	TCGA-38-4629-01A-02T-1207-13	93	TCGA-44-A47F-01A-11H-A24G-13
44	TCGA-38-4630-01A-01T-1207-13	94	TCGA-44-A47G-01A-21H-A24G-13
45	TCGA-38-4631-01A-01T-1754-13	95	TCGA-44-A4SS-01A-11H-A24S-13
46	TCGA-38-4632-01A-01T-1754-13	96	TCGA-44-A4SU-01A-11H-A24S-13
47	TCGA-38-6178-01A-11H-1754-13	97	TCGA-49-4486-01A-01T-1207-13
48	TCGA-38-7271-01A-11H-2038-13	98	TCGA-49-4487-01A-21H-1857-13
49	TCGA-38-A44F-01A-11H-A24G-13	99	TCGA-49-4488-01A-01T-1754-13
50	TCGA-44-2655-01A-01T-0947-13	100	TCGA-49-4490-01A-21H-1857-13

order	sample_id	order	sample_id
101	TCGA-49-4494-01A-01T-1207-13	151	TCGA-55-1594-01A-01T-0947-13
102	TCGA-49-4501-01A-01T-1207-13	152	TCGA-55-1595-01A-01T-0947-13
103	TCGA-49-4505-01A-01T-1207-13	153	TCGA-55-1596-01A-01T-0947-13
104	TCGA-49-4506-01A-01T-1207-13	154	TCGA-55-5899-01A-11H-1627-13
105	TCGA-49-4507-01A-01T-1207-13	155	TCGA-55-6543-01A-11H-1754-13
106	TCGA-49-4510-01A-01T-1207-13	156	TCGA-55-6642-01A-11H-1857-13
107	TCGA-49-4512-01A-21H-1857-13	157	TCGA-55-6712-01A-11H-1857-13
108	TCGA-49-4514-01A-21H-1857-13	158	TCGA-55-6968-01A-11H-1948-13
109	TCGA-49-6742-01A-11H-1857-13	159	TCGA-55-6969-01A-11H-1948-13
110	TCGA-49-6743-01A-11H-1857-13	160	TCGA-55-6970-01A-11H-1948-13
111	TCGA-49-6744-01A-11H-1857-13	161	TCGA-55-6971-01A-11H-1948-13
112	TCGA-49-6745-01A-11H-1857-13	162	TCGA-55-6972-01A-11H-1948-13
113	TCGA-49-6761-01A-31H-1948-13	163	TCGA-55-6978-01A-11H-1948-13
114	TCGA-49-6767-01A-11H-1857-13	164	TCGA-55-6979-01A-11H-1948-13
115	TCGA-50-5044-01A-21H-1857-13	165	TCGA-55-6980-01A-11H-1948-13
116	TCGA-50-5045-01A-01T-1627-13	166	TCGA-55-6981-01A-11H-1948-13
117	TCGA-50-5049-01A-01T-1627-13	167	TCGA-55-6982-01A-11H-1948-13
118	TCGA-50-5051-01A-21H-1857-13	168	TCGA-55-6983-01A-11H-1948-13
119	TCGA-50-5055-01A-01T-1627-13	169	TCGA-55-6984-01A-11H-1948-13
120	TCGA-50-5066-01A-01T-1627-13	170	TCGA-55-6985-01A-11H-1948-13
121	TCGA-50-5068-01A-01T-1627-13	171	TCGA-55-6986-01A-11H-1948-13
122	TCGA-50-5072-01A-21H-1857-13	172	TCGA-55-6987-01A-11H-1948-13
123	TCGA-50-5930-01A-11H-1754-13	173	TCGA-55-7227-01A-11H-2038-13
124	TCGA-50-5931-01A-11H-1754-13	174	TCGA-55-7281-01A-11H-2038-13
125	TCGA-50-5932-01A-11H-1754-13	175	TCGA-55-7283-01A-11H-2038-13
126	TCGA-50-5933-01A-11H-1754-13	176	TCGA-55-7284-01B-11H-2240-13
127	TCGA-50-5935-01A-11H-1754-13	177	TCGA-55-7570-01A-11H-2038-13
128	TCGA-50-5936-01A-11H-1627-13	178	TCGA-55-7573-01A-11H-2038-13
129	TCGA-50-5939-01A-11H-1627-13	179	TCGA-55-7574-01A-11H-2038-13
130	TCGA-50-5941-01A-11H-1754-13	180	TCGA-55-7576-01A-11H-2065-13
131	TCGA-50-5942-01A-21H-1754-13	181	TCGA-55-7724-01A-11H-2169-13
132	TCGA-50-5944-01A-11H-1754-13	182	TCGA-55-7725-01A-11H-2169-13
133	TCGA-50-5946-01A-11H-1754-13	183	TCGA-55-7726-01A-11H-2169-13
134	TCGA-50-6590-01A-12H-1857-13	184	TCGA-55-7727-01A-11H-2169-13
135	TCGA-50-6591-01A-11H-1754-13	185	TCGA-55-7728-01A-11H-2186-13
136	TCGA-50-6592-01A-11H-1754-13	186	TCGA-55-7815-01A-11H-2169-13
137	TCGA-50-6593-01A-11H-1754-13	187	TCGA-55-7816-01A-11H-2169-13
138	TCGA-50-6594-01A-11H-1754-13	188	TCGA-55-7903-01A-11H-2169-13
139	TCGA-50-6595-01A-12H-1857-13	189	TCGA-55-7907-01A-11H-2169-13
140	TCGA-50-6597-01A-11H-1857-13	190	TCGA-55-7910-01A-11H-2169-13
141	TCGA-50-6673-01A-11H-1948-13	191	TCGA-55-7911-01A-11H-2169-13
142	TCGA-50-7109-01A-11H-2038-13	192	TCGA-55-7913-01B-11H-2240-13
143	TCGA-50-8457-01A-11H-2325-13	193	TCGA-55-7914-01A-11H-2169-13
144	TCGA-50-8459-01A-11H-2325-13	194	TCGA-55-7994-01A-11H-2186-13
145	TCGA-50-8460-01A-11H-2325-13	195	TCGA-55-7995-01A-11H-2186-13
146	TCGA-53-7624-01A-11H-2065-13	196	TCGA-55-8085-01A-11H-2240-13
147	TCGA-53-7626-01A-12H-2065-13	197	TCGA-55-8087-01A-11H-2240-13
148	TCGA-53-7813-01A-11H-2169-13	198	TCGA-55-8089-01A-11H-2240-13
149	TCGA-53-A4EZ-01A-12H-A24S-13	199	TCGA-55-8090-01A-11H-2240-13
150	TCGA-55-1592-01A-01T-0947-13	200	TCGA-55-8091-01A-11H-2240-13

order	sample_id	order	sample_id
201	TCGA-55-8092-01A-11H-2240-13	251	TCGA-62-A46Y-01A-11H-A24G-13
202	TCGA-55-8094-01A-11H-2240-13	252	TCGA-62-A470-01A-11H-A24G-13
203	TCGA-55-8096-01A-11H-2240-13	253	TCGA-62-A471-01A-12H-A24G-13
204	TCGA-55-8097-01A-11H-2240-13	254	TCGA-62-A472-01A-11H-A24G-13
205	TCGA-55-8203-01A-11H-2240-13	255	TCGA-64-1676-01A-01T-0947-13
206	TCGA-55-8204-01A-11H-2240-13	256	TCGA-64-1677-01A-01T-0947-13
207	TCGA-55-8205-01A-11H-2240-13	257	TCGA-64-1678-01A-01T-0947-13
208	TCGA-55-8206-01A-11H-2240-13	258	TCGA-64-1679-01A-21H-2065-13
209	TCGA-55-8207-01A-11H-2240-13	259	TCGA-64-1680-01A-02T-0947-13
210	TCGA-55-8208-01A-11H-2240-13	260	TCGA-64-1681-01A-11H-2065-13
211	TCGA-55-8299-01A-11H-2286-13	261	TCGA-64-5774-01A-01T-1627-13
212	TCGA-55-8301-01A-11H-2286-13	262	TCGA-64-5775-01A-01T-1627-13
213	TCGA-55-8302-01A-11H-2325-13	263	TCGA-64-5778-01A-01T-1627-13
214	TCGA-55-8505-01A-11H-2402-13	264	TCGA-64-5779-01A-01T-1627-13
215	TCGA-55-8506-01A-11H-2402-13	265	TCGA-64-5781-01A-01T-1627-13
216	TCGA-55-8507-01A-11H-2402-13	266	TCGA-64-5815-01A-01T-1627-13
217	TCGA-55-8508-01A-11H-2402-13	267	TCGA-67-3770-01A-01T-0947-13
218	TCGA-55-8510-01A-11H-2402-13	268	TCGA-67-3771-01A-01T-0947-13
219	TCGA-55-8511-01A-11H-2402-13	269	TCGA-67-3772-01A-01T-0947-13
220	TCGA-55-8512-01A-11H-2402-13	270	TCGA-67-3773-01A-01T-0947-13
221	TCGA-55-8513-01A-11H-2402-13	271	TCGA-67-3774-01A-01T-0947-13
222	TCGA-55-8514-01A-11H-2402-13	272	TCGA-67-4679-01B-01T-1754-13
223	TCGA-55-8614-01A-11H-2402-13	273	TCGA-67-6215-01A-11H-1754-13
224	TCGA-55-8615-01A-11H-2402-13	274	TCGA-67-6216-01A-11H-1754-13
225	TCGA-55-8616-01A-11H-2402-13	275	TCGA-67-6217-01A-11H-1754-13
226	TCGA-55-8619-01A-11H-2402-13	276	TCGA-69-7760-01A-11H-2169-13
227	TCGA-55-8620-01A-11H-2402-13	277	TCGA-69-7761-01A-11H-2169-13
228	TCGA-55-8621-01A-11H-2402-13	278	TCGA-69-7763-01A-11H-2169-13
229	TCGA-55-A48X-01A-11H-A24G-13	279	TCGA-69-7764-01A-11H-2169-13
230	TCGA-55-A48Y-01A-11H-A24G-13	280	TCGA-69-7765-01A-11H-2169-13
231	TCGA-55-A48Z-01A-12H-A24S-13	281	TCGA-69-7973-01A-11H-2186-13
232	TCGA-55-A490-01A-11H-A24G-13	282	TCGA-69-7974-01A-11H-2186-13
233	TCGA-55-A491-01A-11H-A24G-13	283	TCGA-69-7978-01A-11H-2186-13
234	TCGA-55-A492-01A-11H-A24G-13	284	TCGA-69-7979-01A-11H-2186-13
235	TCGA-55-A493-01A-11H-A24G-13	285	TCGA-69-7980-01A-11H-2186-13
236	TCGA-55-A494-01A-11H-A24S-13	286	TCGA-69-8253-01A-11H-2286-13
237	TCGA-55-A4DF-01A-11H-A24G-13	287	TCGA-69-8254-01A-11H-2286-13
238	TCGA-55-A4DG-01A-11H-A24G-13	288	TCGA-69-8255-01A-11H-2286-13
239	TCGA-62-8394-01A-11H-2325-13	289	TCGA-69-8453-01A-12H-2325-13
240	TCGA-62-8395-01A-11H-2325-13	290	TCGA-69-A59K-01A-11H-A263-13
241	TCGA-62-8397-01A-11H-2325-13	291	TCGA-71-6725-01A-11H-1857-13
242	TCGA-62-8398-01A-11H-2325-13	292	TCGA-71-8520-01A-11H-2402-13
243	TCGA-62-8399-01A-21H-2325-13	293	TCGA-73-4658-01A-01T-1754-13
244	TCGA-62-8402-01A-11H-2325-13	294	TCGA-73-4659-01A-01T-1207-13
245	TCGA-62-A46O-01A-11H-A24G-13	295	TCGA-73-4662-01A-01T-1207-13
246	TCGA-62-A46P-01A-11H-A24G-13	296	TCGA-73-4675-01A-01T-1207-13
247	TCGA-62-A46R-01A-11H-A24G-13	297	TCGA-73-4676-01A-01T-1754-13
248	TCGA-62-A46S-01A-11H-A24G-13	298	TCGA-73-7498-01A-12H-2186-13
249	TCGA-62-A46U-01A-11H-A24G-13	299	TCGA-73-7499-01A-11H-2186-13
250	TCGA-62-A46V-01A-11H-A24G-13	300	TCGA-75-5122-01A-01T-1754-13

order	sample_id	order	sample_id
301	TCGA-75-5125-01A-01T-1754-13	351	TCGA-80-5611-01A-01T-1627-13
302	TCGA-75-5126-01A-01T-1754-13	352	TCGA-83-5908-01A-21H-2286-13
303	TCGA-75-5146-01A-01T-1627-13	353	TCGA-86-6562-01A-11H-1754-13
304	TCGA-75-5147-01A-01T-1627-13	354	TCGA-86-6851-01A-11H-1948-13
305	TCGA-75-6203-01A-11H-1754-13	355	TCGA-86-7701-01A-11H-2169-13
306	TCGA-75-6205-01A-11H-1754-13	356	TCGA-86-7711-01A-11H-2065-13
307	TCGA-75-6206-01A-11H-1754-13	357	TCGA-86-7713-01A-11H-2065-13
308	TCGA-75-6207-01A-11H-1754-13	358	TCGA-86-7714-01A-12H-2169-13
309	TCGA-75-6211-01A-11H-1754-13	359	TCGA-86-7953-01A-11H-2186-13
310	TCGA-75-6212-01A-11H-1754-13	360	TCGA-86-7954-01A-11H-2186-13
311	TCGA-75-6214-01A-41H-1948-13	361	TCGA-86-7955-01A-11H-2186-13
312	TCGA-75-7025-01A-12H-1948-13	362	TCGA-86-8054-01A-11H-2240-13
313	TCGA-75-7027-01A-11H-1948-13	363	TCGA-86-8055-01A-11H-2240-13
314	TCGA-75-7030-01A-11H-1948-13	364	TCGA-86-8056-01A-11H-2240-13
315	TCGA-75-7031-01A-11H-1948-13	365	TCGA-86-8073-01A-11H-2240-13
316	TCGA-78-7143-01A-11H-2038-13	366	TCGA-86-8074-01A-11H-2240-13
317	TCGA-78-7145-01A-11H-2038-13	367	TCGA-86-8075-01A-11H-2240-13
318	TCGA-78-7146-01A-11H-2038-13	368	TCGA-86-8076-01A-31H-2240-13
319	TCGA-78-7147-01A-11H-2038-13	369	TCGA-86-8278-01A-11H-2286-13
320	TCGA-78-7148-01A-11H-2038-13	370	TCGA-86-8279-01A-11H-2286-13
321	TCGA-78-7149-01A-11H-2038-13	371	TCGA-86-8280-01A-11H-2286-13
322	TCGA-78-7150-01A-21H-2038-13	372	TCGA-86-8281-01A-11H-2286-13
323	TCGA-78-7152-01A-11H-2038-13	373	TCGA-86-8358-01A-11H-2325-13
324	TCGA-78-7153-01A-11H-2038-13	374	TCGA-86-8359-01A-11H-2325-13
325	TCGA-78-7154-01A-11H-2038-13	375	TCGA-86-8585-01A-11H-2402-13
326	TCGA-78-7155-01A-11H-2038-13	376	TCGA-86-8668-01A-11H-2402-13
327	TCGA-78-7156-01A-11H-2038-13	377	TCGA-86-8669-01A-11H-2402-13
328	TCGA-78-7158-01A-11H-2038-13	378	TCGA-86-8671-01A-11H-2402-13
329	TCGA-78-7159-01A-11H-2038-13	379	TCGA-86-8672-01A-21H-2402-13
330	TCGA-78-7160-01A-11H-2038-13	380	TCGA-86-8673-01A-11H-2402-13
331	TCGA-78-7161-01A-11H-2038-13	381	TCGA-86-8674-01A-21H-2402-13
332	TCGA-78-7162-01A-21H-2065-13	382	TCGA-86-A456-01A-11H-A24G-13
333	TCGA-78-7163-01A-12H-2065-13	383	TCGA-86-A4D0-01A-11H-A24G-13
334	TCGA-78-7166-01A-12H-2065-13	384	TCGA-86-A4JF-01A-11H-A24S-13
335	TCGA-78-7167-01A-11H-2065-13	385	TCGA-86-A4P7-01A-11H-A24S-13
336	TCGA-78-7220-01A-11H-2038-13	386	TCGA-86-A4P8-01A-11H-A24S-13
337	TCGA-78-7535-01A-11H-2065-13	387	TCGA-91-6828-01A-11H-1857-13
338	TCGA-78-7536-01A-11H-2065-13	388	TCGA-91-6829-01A-21H-1857-13
339	TCGA-78-7537-01A-11H-2065-13	389	TCGA-91-6830-01A-11H-1948-13
340	TCGA-78-7539-01A-11H-2065-13	390	TCGA-91-6831-01A-11H-1857-13
341	TCGA-78-7540-01A-11H-2065-13	391	TCGA-91-6835-01A-11H-1857-13
342	TCGA-78-7542-01A-21H-2065-13	392	TCGA-91-6836-01A-21H-1857-13
343	TCGA-78-7633-01A-11H-2065-13	393	TCGA-91-6840-01A-11H-1948-13
344	TCGA-78-8640-01A-11H-2402-13	394	TCGA-91-6847-01A-11H-1948-13
345	TCGA-78-8648-01A-11H-2402-13	395	TCGA-91-6848-01A-11H-1948-13
346	TCGA-78-8655-01A-11H-2402-13	396	TCGA-91-6849-01A-11H-1948-13
347	TCGA-78-8660-01A-11H-2402-13	397	TCGA-91-7771-01A-11H-2169-13
348	TCGA-78-8662-01A-11H-2402-13	398	TCGA-91-8496-01A-11H-2402-13
349	TCGA-80-5607-01A-31H-1948-13	399	TCGA-91-8497-01A-11H-2402-13
350	TCGA-80-5608-01A-31H-1948-13	400	TCGA-91-8499-01A-11H-2402-13

order	sample_id	order	sample_id
401	TCGA-91-A4BC-01A-11R-A24G-13	451	TCGA-99-8033-01A-11H-2240-13
402	TCGA-91-A4BD-01A-11R-A24G-13	452	TCGA-J2-8192-01A-11H-2240-13
403	TCGA-93-7347-01A-11H-2186-13	453	TCGA-J2-8194-01A-11H-2240-13
404	TCGA-93-7348-01A-21H-2038-13	454	TCGA-J2-A4AD-01A-11R-A24G-13
405	TCGA-93-8067-01A-11H-2286-13	455	TCGA-J2-A4AE-01A-21R-A24G-13
406	TCGA-93-A4JN-01A-11H-A24S-13	456	TCGA-J2-A4AG-01A-11H-A24G-13
407	TCGA-93-A4JO-01A-21H-A24S-13	457	TCGA-L4-A4E5-01A-11H-A24S-13
408	TCGA-93-A4JP-01A-11H-A24S-13	458	TCGA-L4-A4E6-01A-11R-A24G-13
409	TCGA-93-A4JQ-01A-11H-A24S-13	459	TCGA-L9-A443-01A-12H-A24G-13
410	TCGA-95-7039-01A-11H-1948-13	460	TCGA-L9-A444-01A-21H-A24G-13
411	TCGA-95-7043-01A-11H-1948-13	461	TCGA-MN-A4N1-01A-11H-A24S-13
412	TCGA-95-7562-01A-11H-2240-13	462	TCGA-MN-A4N4-01A-12H-A24S-13
413	TCGA-95-7567-01A-11H-2065-13	463	TCGA-MN-A4N5-01A-11H-A24S-13
414	TCGA-95-7944-01A-11H-2186-13	464	TCGA-MP-A4SV-01A-11H-A24S-13
415	TCGA-95-7947-01A-11H-2186-13	465	TCGA-MP-A4SW-01A-21H-A24S-13
416	TCGA-95-7948-01A-11H-2186-13	466	TCGA-MP-A4SY-01A-21H-A24S-13
417	TCGA-95-8039-01A-11H-2240-13	467	TCGA-MP-A4T2-01A-11H-A24S-13
418	TCGA-95-8494-01A-11H-2325-13	468	TCGA-MP-A4T4-01A-11H-A263-13
419	TCGA-95-A4VK-01A-11H-A263-13	469	TCGA-MP-A4T6-01A-32H-A263-13
420	TCGA-95-A4VN-01A-11H-A263-13	470	TCGA-MP-A4T7-01A-11H-A24S-13
421	TCGA-95-A4VP-01A-21H-A263-13	471	TCGA-MP-A4T8-01A-11H-A24S-13
422	TCGA-97-7546-01A-11H-2038-13	472	TCGA-MP-A4T9-01A-11H-A24S-13
423	TCGA-97-7547-01A-11H-2038-13	473	TCGA-MP-A4TA-01A-21H-A24S-13
424	TCGA-97-7552-01A-11H-2038-13	474	TCGA-MP-A4TC-01A-11H-A24S-13
425	TCGA-97-7553-01A-21H-2038-13	475	TCGA-MP-A4TD-01A-32H-A263-13
426	TCGA-97-7554-01A-11H-2038-13	476	TCGA-MP-A4TE-01A-22H-A263-13
427	TCGA-97-7937-01A-11H-2169-13	477	TCGA-MP-A4TF-01A-11H-A263-13
428	TCGA-97-7938-01A-11H-2169-13	478	TCGA-MP-A4TH-01A-31H-A263-13
429	TCGA-97-7941-01A-11H-2186-13	479	TCGA-MP-A4TI-01A-21H-A24S-13
430	TCGA-97-8171-01A-11H-2286-13	480	TCGA-MP-A4TJ-01A-51H-A263-13
431	TCGA-97-8172-01A-11H-2286-13	481	TCGA-MP-A4TK-01A-11H-A24S-13
432	TCGA-97-8174-01A-11H-2286-13	482	TCGA-MP-A5C7-01A-11H-A263-13
433	TCGA-97-8175-01A-11H-2286-13	483	TCGA-NJ-A4YF-01A-12H-A263-13
434	TCGA-97-8176-01A-11H-2402-13	484	TCGA-NJ-A4YG-01A-22H-A263-13
435	TCGA-97-8177-01A-11H-2286-13	485	TCGA-NJ-A4YI-01A-11H-A263-13
436	TCGA-97-8179-01A-11H-2286-13	486	TCGA-NJ-A4YP-01A-11H-A263-13
437	TCGA-97-8547-01A-11H-2402-13	487	TCGA-NJ-A4YQ-01A-11H-A263-13
438	TCGA-97-8552-01A-11H-2402-13	488	TCGA-NJ-A55A-01A-11H-A263-13
439	TCGA-97-A4LX-01A-11H-A24S-13	489	TCGA-NJ-A55O-01A-11H-A263-13
440	TCGA-97-A4M0-01A-11H-A24S-13	490	TCGA-NJ-A55R-01A-11H-A263-13
441	TCGA-97-A4M1-01A-11H-A24S-13	491	TCGA-O1-A52J-01A-11H-A263-13
442	TCGA-97-A4M2-01A-12H-A24S-13		
443	TCGA-97-A4M3-01A-11H-A24S-13		
444	TCGA-97-A4M5-01A-11H-A24S-13		
445	TCGA-97-A4M6-01A-11H-A24S-13		
446	TCGA-97-A4M7-01A-11H-A24S-13		
447	TCGA-99-7458-01A-11H-2038-13		
448	TCGA-99-8025-01A-11H-2240-13		
449	TCGA-99-8028-01A-11H-2240-13		
450	TCGA-99-8032-01A-11H-2240-13		
