# Supplementary material for: deGPS is a powerful tool for detecting differential expression in RNA-sequencing studies
Source: BMC Genomics. 2015 Jun 13;16(1):455. doi: 10.1186/s12864-015-1676-0 (PMC4465298; doi:10.1186/s12864-015-1676-0)
Supplement: Additional file 2: Table S2. — -TCGA samples used in mRNA-Seq simulations. [file 12864_2015_1676_MOESM2_ESM.pdf]

Table S2 TCGA samples used in mRNA-Seq simulations.

order	sample_id	order	sample_id
1	TCGA-38-4625-11A-01R-1758-07	51	TCGA-L9-A443-01A-12R-A24H-07
2	TCGA-38-4626-11A-01R-1758-07	52	TCGA-62-A46O-01A-11R-A24H-07
3	TCGA-38-4627-11A-01R-1758-07	53	TCGA-62-A46P-01A-11R-A24H-07
4	TCGA-38-4632-11A-01R-1755-07	54	TCGA-93-A4JO-01A-21R-A24X-07
5	TCGA-44-2655-11A-01R-1758-07	55	TCGA-97-A4M3-01A-11R-A24X-07
6	TCGA-44-2657-11A-01R-1758-07	56	TCGA-97-A4M2-01A-12R-A24X-07
7	TCGA-44-2661-11A-01R-1758-07	57	TCGA-97-A4M7-01A-11R-A24X-07
8	TCGA-44-2662-11A-01R-1758-07	58	TCGA-97-A4LX-01A-11R-A24X-07
9	TCGA-44-2665-11A-01R-1758-07	59	TCGA-50-5933-11A-01R-1755-07
10	TCGA-44-2668-11A-01R-1758-07	60	TCGA-93-A4JQ-01A-11R-A24X-07
11	TCGA-44-3396-11A-01R-1758-07	61	TCGA-MN-A4N1-01A-11R-A24X-07
12	TCGA-44-5645-11A-01R-1628-07	62	TCGA-L4-A4E5-01A-11R-A24X-07
13	TCGA-44-6144-11A-01R-1755-07	63	TCGA-97-A4M0-01A-11R-A24X-07
14	TCGA-44-6145-11A-01R-1858-07	64	TCGA-55-A494-01A-11R-A24X-07
15	TCGA-44-6146-11A-01R-1858-07	65	TCGA-MP-A4T9-01A-11R-A24X-07
16	TCGA-44-6147-11A-01R-1858-07	66	TCGA-MN-A4N4-01A-12R-A24X-07
17	TCGA-44-6148-11A-01R-1858-07	67	TCGA-MP-A4SW-01A-21R-A24X-07
18	TCGA-44-6776-11A-01R-1858-07	68	TCGA-93-A4JN-01A-11R-A24X-07
19	TCGA-44-6778-11A-01R-1858-07	69	TCGA-55-A48Z-01A-12R-A24X-07
20	TCGA-49-4490-11A-01R-1858-07	70	TCGA-MP-A4TK-01A-11R-A24X-07
21	TCGA-49-4512-11A-01R-1858-07	71	TCGA-44-A4SS-01A-11R-A24X-07
22	TCGA-49-6742-11A-01R-1858-07	72	TCGA-55-6971-11A-01R-1949-07
23	TCGA-49-6743-11A-01R-1858-07	73	TCGA-93-A4JP-01A-11R-A24X-07
24	TCGA-49-6761-11A-01R-1949-07	74	TCGA-55-6970-11A-01R-1949-07
25	TCGA-50-5930-11A-01R-1755-07	75	TCGA-97-A4M6-01A-11R-A24X-07
26	TCGA-50-5931-11A-01R-1858-07	76	TCGA-49-6745-11A-01R-1858-07
27	TCGA-50-5935-11A-01R-1858-07	77	TCGA-MP-A4TA-01A-21R-A24X-07
28	TCGA-50-5936-11A-01R-1628-07	78	TCGA-MN-A4N5-01A-11R-A24X-07
29	TCGA-50-5939-11A-01R-1628-07	79	TCGA-MP-A4SY-01A-21R-A24X-07
30	TCGA-50-6595-11A-01R-1858-07	80	TCGA-55-A492-01A-11R-A24H-07
31	TCGA-55-6968-11A-01R-1949-07	81	TCGA-MP-A4T7-01A-11R-A24X-07
32	TCGA-55-6969-11A-01R-1949-07	82	TCGA-53-A4EZ-01A-12R-A24X-07
33	TCGA-55-6972-11A-01R-1949-07	83	TCGA-62-A46R-01A-11R-A24H-07
34	TCGA-55-6975-11A-01R-1949-07	84	TCGA-91-6849-11A-01R-1949-07
35	TCGA-55-6978-11A-01R-1949-07	85	TCGA-86-A4P7-01A-11R-A24X-07
36	TCGA-55-6979-11A-01R-1949-07	86	TCGA-44-6777-11A-01R-1858-07
37	TCGA-55-6980-11A-01R-1949-07	87	TCGA-MP-A4T8-01A-11R-A24X-07
38	TCGA-55-6981-11A-01R-1949-07	88	TCGA-86-A4JF-01A-11R-A24X-07
39	TCGA-55-6982-11A-01R-1949-07	89	TCGA-55-A4DG-01A-11R-A24H-07
40	TCGA-55-6983-11A-01R-1949-07	90	TCGA-MP-A4SV-01A-11R-A24X-07
41	TCGA-55-6984-11A-01R-1949-07	91	TCGA-86-A4P8-01A-11R-A24X-07
42	TCGA-55-6985-11A-01R-1949-07	92	TCGA-L9-A444-01A-21R-A24H-07
43	TCGA-55-6986-11A-01R-1949-07	93	TCGA-MP-A4TI-01A-21R-A24X-07
44	TCGA-73-4676-11A-01R-1755-07	94	TCGA-50-5932-11A-01R-1755-07
45	TCGA-91-6828-11A-01R-1858-07	95	TCGA-L4-A4E6-01A-11R-A24H-07
46	TCGA-91-6829-11A-01R-1858-07	96	TCGA-49-6744-11A-01R-1858-07
47	TCGA-91-6831-11A-02R-1858-07	97	TCGA-97-A4M1-01A-11R-A24X-07
48	TCGA-91-6835-11A-01R-1858-07	98	TCGA-MP-A4TC-01A-11R-A24X-07
49	TCGA-91-6836-11A-01R-1858-07	99	TCGA-44-A4SU-01A-11R-A24X-07
50	TCGA-91-6847-11A-01R-1949-07	100	TCGA-97-A4M5-01A-11R-A24X-07

Note: TCGA-62-A46O is used for plotting Figure 1.
