# Supplementary material for: deGPS is a powerful tool for detecting differential expression in RNA-sequencing studies
Source: BMC Genomics. 2015 Jun 13;16(1):455. doi: 10.1186/s12864-015-1676-0 (PMC4465298; doi:10.1186/s12864-015-1676-0)
Supplement: Additional file 3: Document S1. — -Simulation settings. [file 12864_2015_1676_MOESM3_ESM.pdf]

Document S1

In real data-driven simulations, to estimate FDR and TPR under alternative hypothesis, we first randomly generated two groups of samples and randomly chose a subset of transcripts. Subsequently, we made two types of changes in the selected transcripts to create differential expression between two groups. In the “shift” transformation, we added normally distributed quantities of read counts (i.e., with small variance and mean of 20, 40, 60, 80, 100, 120, 140, 160, 180, and 200 in miRNA; 50, 100, 150, 500, 3000, and 6000 in mRNA) into the randomly selected transcripts in either group. In the “shift” and “scaling” transformation, we multiplied the selected transcripts by normally distributed quantities of read counts (i.e., with small variance and mean of 2, 1.2, 1.4, 1.7, 1.9, 2.1, 2.3, 2.6, 2.8, and 3 in miRNA; 1.1, 1.2, 1.3, 1.4, 1.5, and 1.6 in mRNA) after applying the “shift” transformation (20 in miRNA and 50 in mRNA).

In comparison with edgeR, one of the top performers of differential analysis of sequence count data, we applied different parameter settings in the R package. Three normalization methods, TMM, relative log expression (RLE) and upper quartile are embedded in the R package (v3.6.7), as well as two types of statistical tests. One is likelihood ratio test (LRT) based on the generalized linear model (GLM), i.e., glmLRT. The other one is glmQLFTest, which is alternative to glmLRT and replaces the Chi-square approximation to the likelihood ratio statistic with a quasi-likelihood F-test. We systematically evaluated different user-defined parameter settings in edgeR:

**Name**	**Normalization**	**DE Test**
edgeR1	TMM	glmLRT
edgeR2	TMM	glmQLFTest
edgeR3	RLE	glmLRT
edgeR4	RLE	glmQLFTest
edgeR5	upperquartile	glmLRT
edgeR6	upperquartile	glmQLFTest

The real data analysis of the developmental transcriptome of Drosophila can be found in our released R package–deGPS (https://github.com/LL-LAB-MCW). compcodeR-based simulations can be repeated by the R codes which are available in **Additional file 15**. Since TCGA data used in our data-driven simulations are extremely large, and simulations are very time-consuming, we are not able to provide codes and associated TCGA data for readers and reviewers to repeat these simulations.
