# Supplementary figures and images for: deGPS is a powerful tool for detecting differential expression in RNA-sequencing studies
Source: BMC Genomics. 2015 Jun 13;16(1):455. doi: 10.1186/s12864-015-1676-0 (PMC4465298; doi:10.1186/s12864-015-1676-0)

**Figure S1. A flowchart for method comparisons in simulations.**

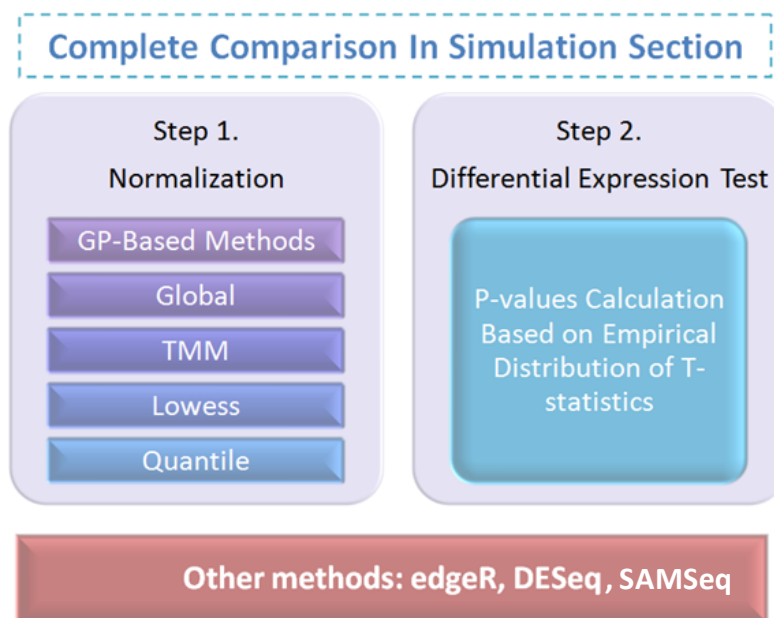

Supplement: Additional file 4: Figure S1. — -A flowchart for method comparisons in simulations. [file 12864_2015_1676_MOESM4_ESM.pdf]
