# Supplementary material for: deGPS is a powerful tool for detecting differential expression in RNA-sequencing studies
Source: BMC Genomics. 2015 Jun 13;16(1):455. doi: 10.1186/s12864-015-1676-0 (PMC4465298; doi:10.1186/s12864-015-1676-0)

**Figure S2. Type I error and false discovery rate of edgeR with different parameter settings.** Different parameter settings in edgeR were defined in **Document S1.** Methods in red font are those inflate type I error and/or false discovery rate.

(**A**) miRNA

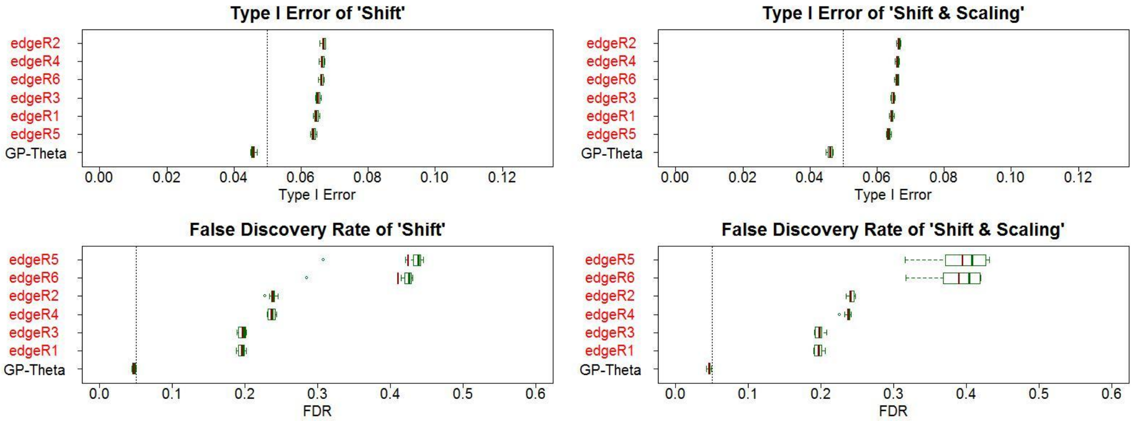


(**B**) mRNA

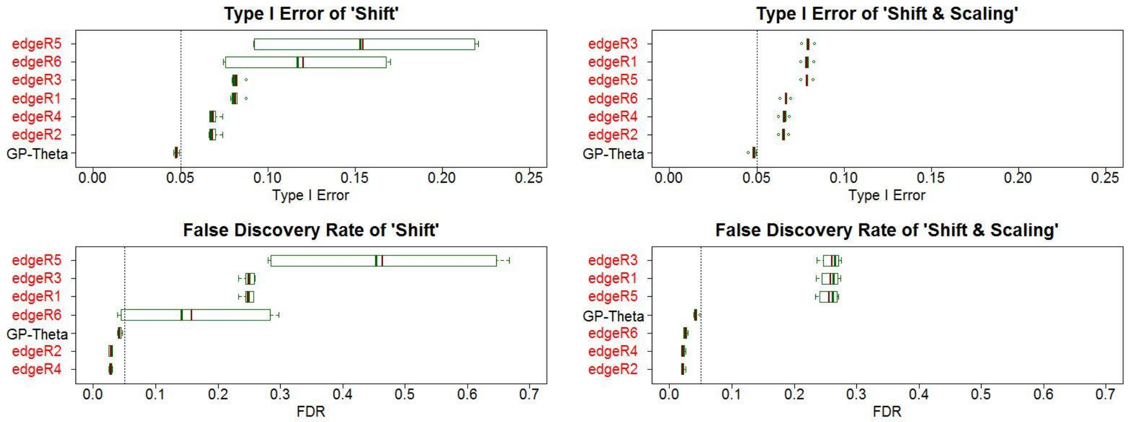

Supplement: Additional file 5: Figure S2. — -Type I error and false discovery rate of edgeR with different parameter settings. Different parameter settings in edgeR were defined in Document S1. Methods in red font are those inflate type I error and/or false discovery rate. [file 12864_2015_1676_MOESM5_ESM.pdf]
