# Supplementary material for: deGPS is a powerful tool for detecting differential expression in RNA-sequencing studies
Source: BMC Genomics. 2015 Jun 13;16(1):455. doi: 10.1186/s12864-015-1676-0 (PMC4465298; doi:10.1186/s12864-015-1676-0)

Figure S3. True positive rate of edgeR with different parameter settings. Methods in red font are those inflate type I error and/or false discovery rate.

(**A**) miRNA

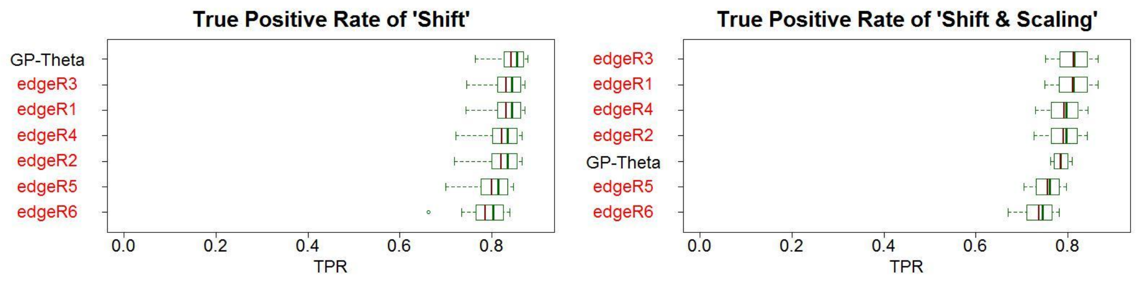

(**B**) mRNA

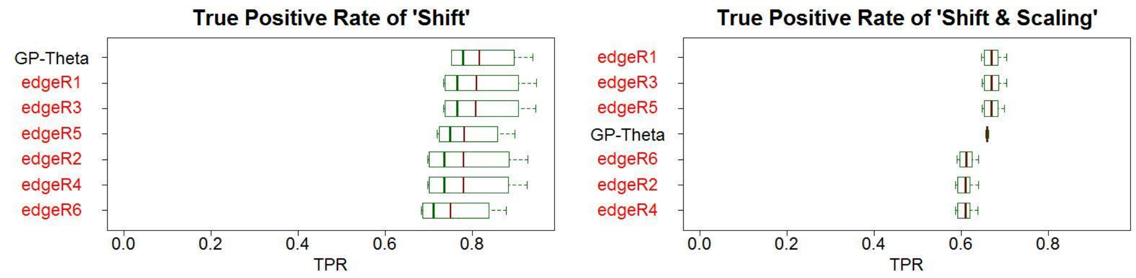

Supplement: Additional file 6: Figure S3. — -True positive rate of edgeR with different parameter settings. Methods in red font are those inflate type I error and/or false discovery rate. [file 12864_2015_1676_MOESM6_ESM.pdf]
