# Supplementary material for: deGPS is a powerful tool for detecting differential expression in RNA-sequencing studies
Source: BMC Genomics. 2015 Jun 13;16(1):455. doi: 10.1186/s12864-015-1676-0 (PMC4465298; doi:10.1186/s12864-015-1676-0)

Figure S5. Simulation results from compcodeR. Sample size was set as 8 subjects per group. Note that edgeR2 does not control correct type I error when increasing sample size although its FDR is at the desired level.

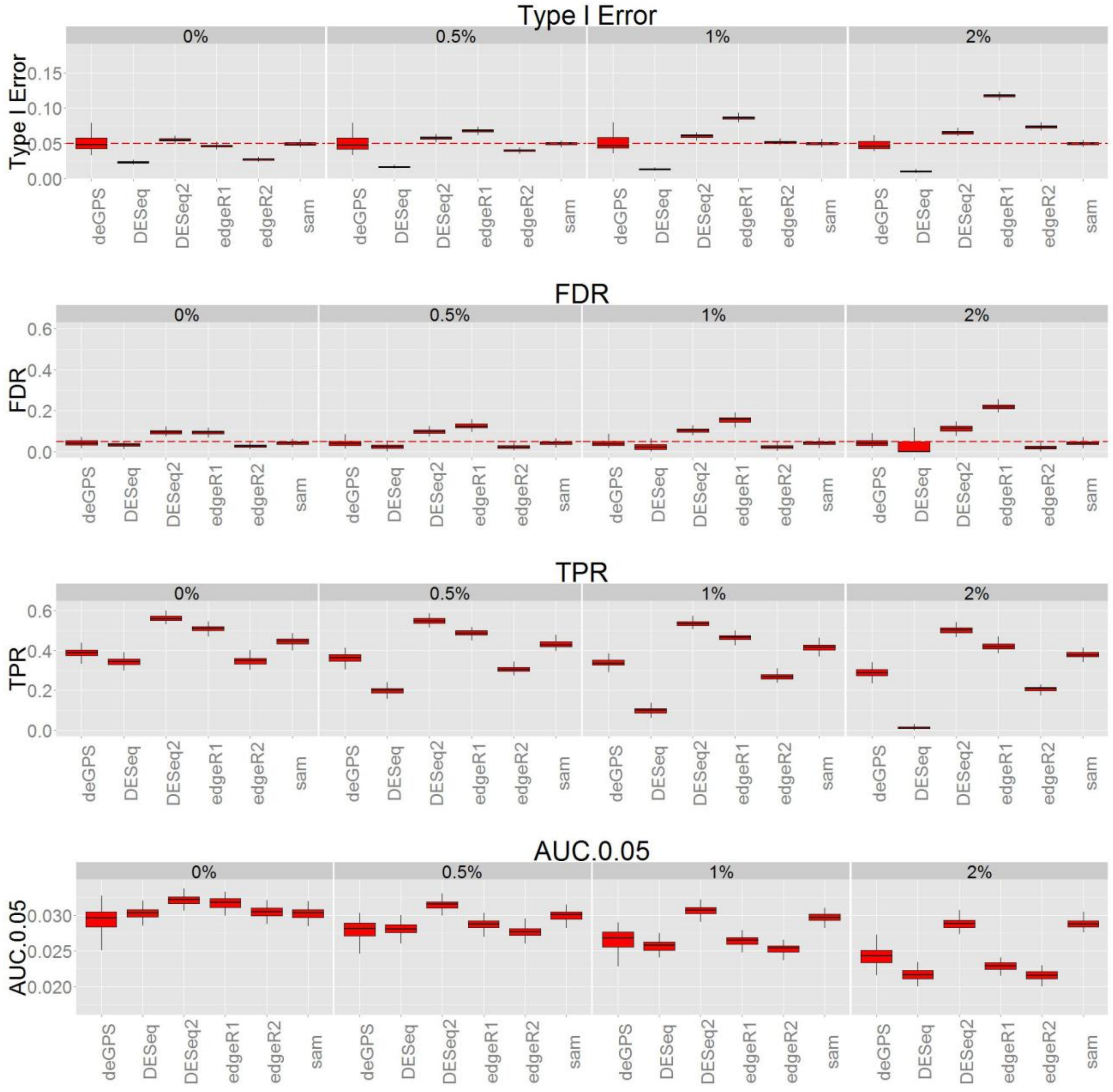

Supplement: Additional file 8: Figure S5. — -Simulation results from compcodeR. Sample size was set as 8 subjects per group. Note that edgeR2 does not control correct type I error when increasing sample size although its FDR is at the desired level. [file 12864_2015_1676_MOESM8_ESM.pdf]
