# Supplementary material for: deGPS is a powerful tool for detecting differential expression in RNA-sequencing studies
Source: BMC Genomics. 2015 Jun 13;16(1):455. doi: 10.1186/s12864-015-1676-0 (PMC4465298; doi:10.1186/s12864-015-1676-0)
Supplement: Additional file 9: Figure S6. — -Effects of different FDR adjustment methods and T-statistics on the performance on deGPS. Sample sizes were set as (A) 5 and (B) 8 subjects per group. The default FDR adjustment of deGPS is BH method; two additional FDR adjustments QVAL and LFDR were evaluated. The default statistic of deGPS is ordinary T-statistic in permutations; regularized T-statistic (st) was evaluated. [file 12864_2015_1676_MOESM9_ESM.pdf]

Figure S6-Effects of different FDR adjustment methods and T-statistics on the performance on deGPS. Sample sizes were set as (A) 5 and (B) 8 subjects per group. The default FDR adjustment of deGPS is BH method; two additional FDR adjustments QVAL and LFDR were evaluated. The default statistic of deGPS is ordinary T-statistic in permutations; regularized T-statistic (st) was evaluated.

(A)

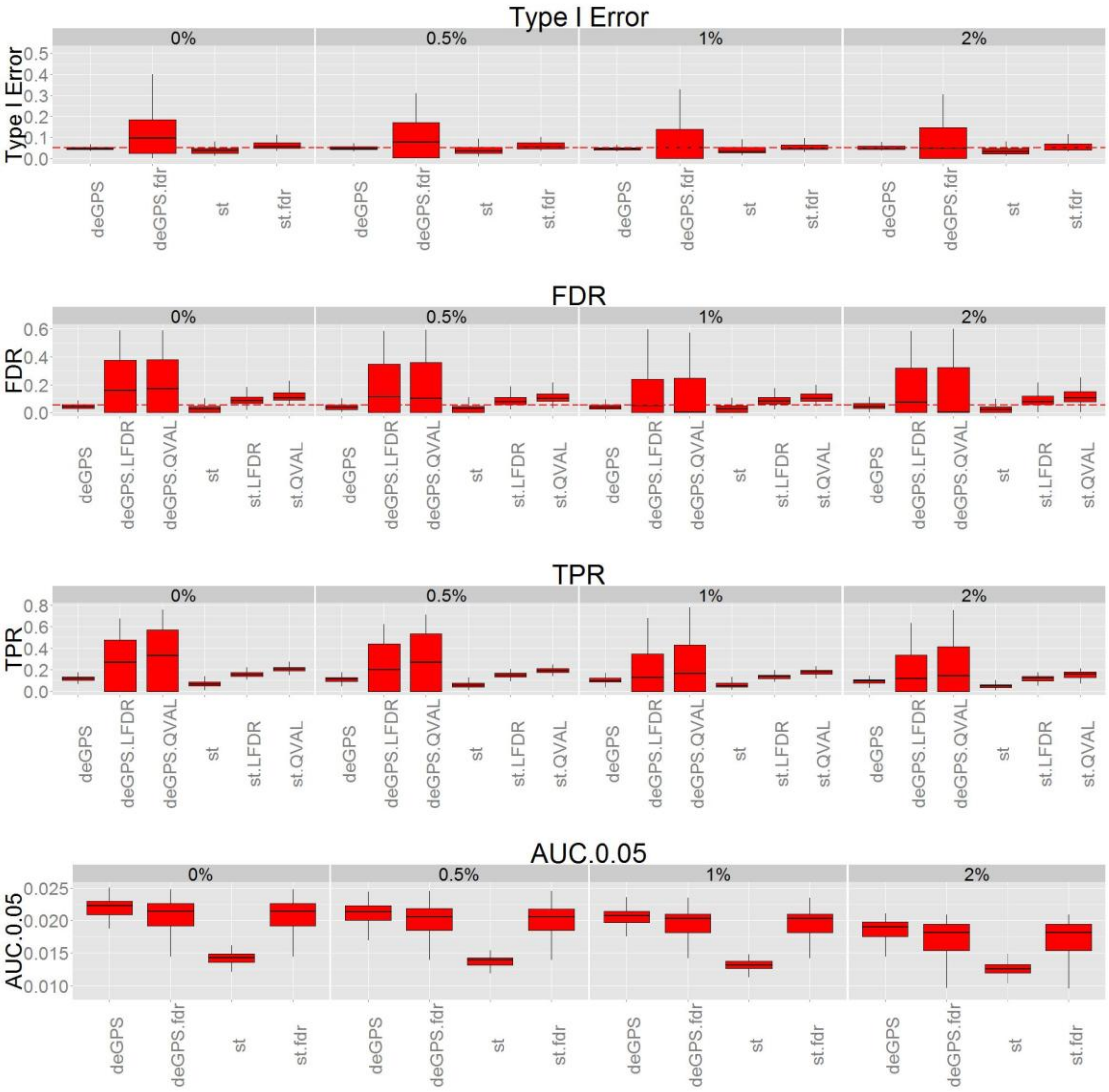

(B)

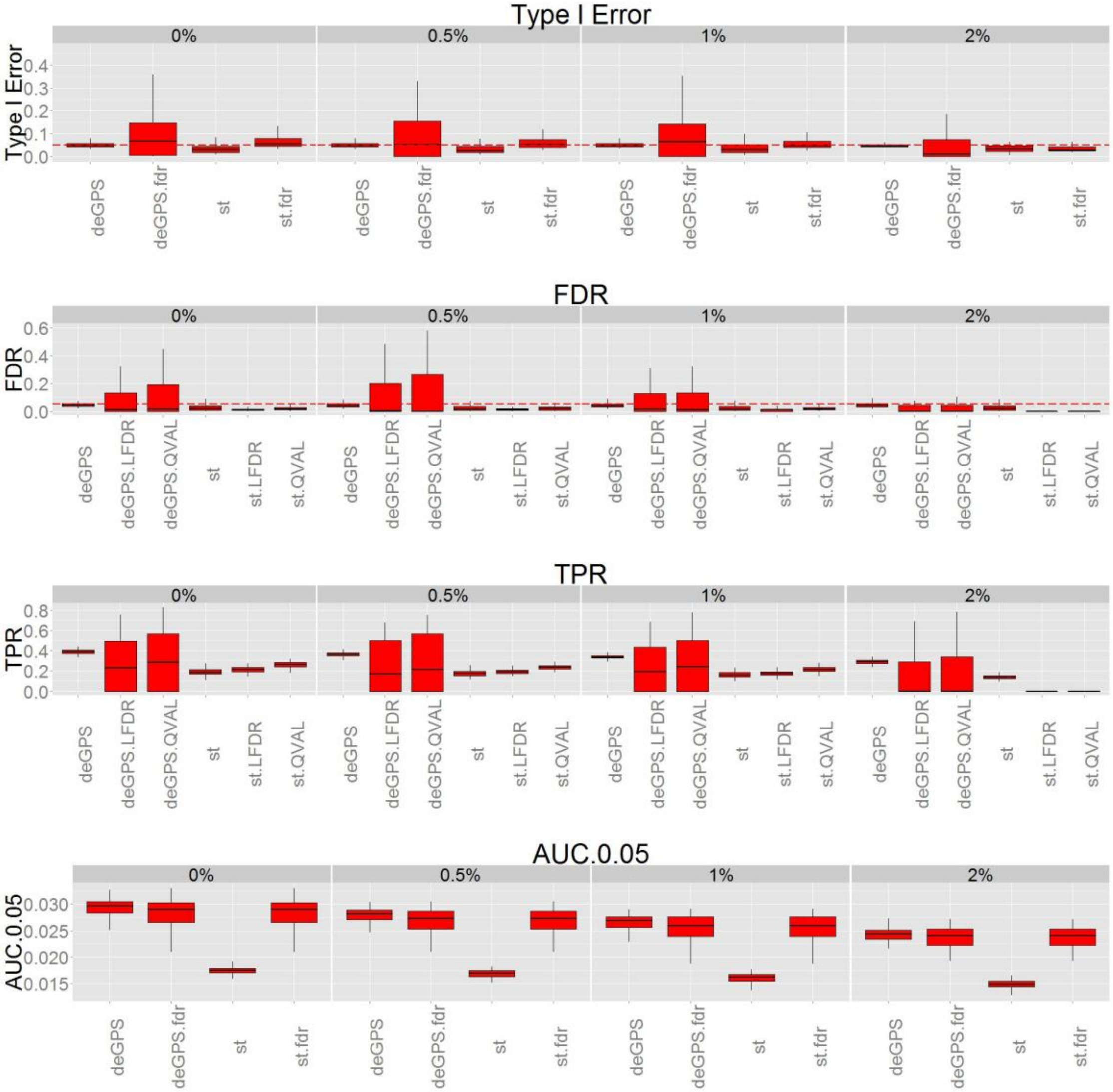
Type I Error
0%
0.5%
1%
2%
Type I Error
0.0
0.1
0.2
0.3
0.4
deGPS
deGPS.fdr
st
st.fdr
FDR
0.0
0.2
0.4
0.6
deGPS
deGPS.LFDR
deGPS.QVAL
st
st.LFDR
st.QVAL
TPR
0.0
0.2
0.4
0.6
0.8
AUC.0.05
0.015
0.020
0.025
0.030
