# Supplementary material for: deGPS is a powerful tool for detecting differential expression in RNA-sequencing studies
Source: BMC Genomics. 2015 Jun 13;16(1):455. doi: 10.1186/s12864-015-1676-0 (PMC4465298; doi:10.1186/s12864-015-1676-0)

Figure S7. Genes differentially expressed between any two non-adjacent developmental stages of Drosophila melanogaster

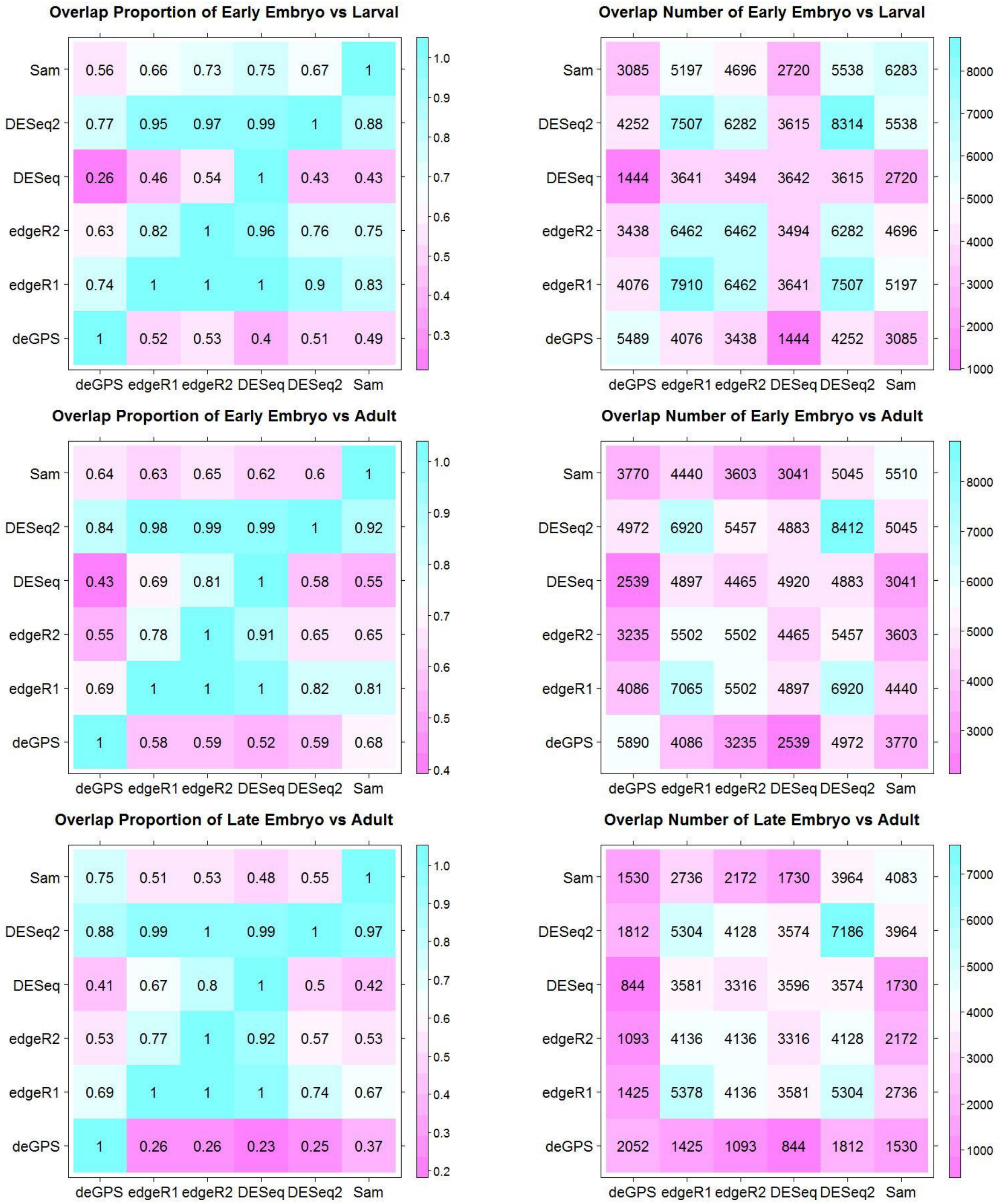

Supplement: Additional file 10: Figure S7. — -Genes differentially expressed between any two non-adjacent developmental stages of Drosophila melanogaster [file 12864_2015_1676_MOESM10_ESM.pdf]
