# Supplementary material for: deGPS is a powerful tool for detecting differential expression in RNA-sequencing studies
Source: BMC Genomics. 2015 Jun 13;16(1):455. doi: 10.1186/s12864-015-1676-0 (PMC4465298; doi:10.1186/s12864-015-1676-0)

**Figure S8. Average false positive number and type I error when comparing two groups that are randomly and equally sampled from different developmental stages of Drosophila melanogaster**

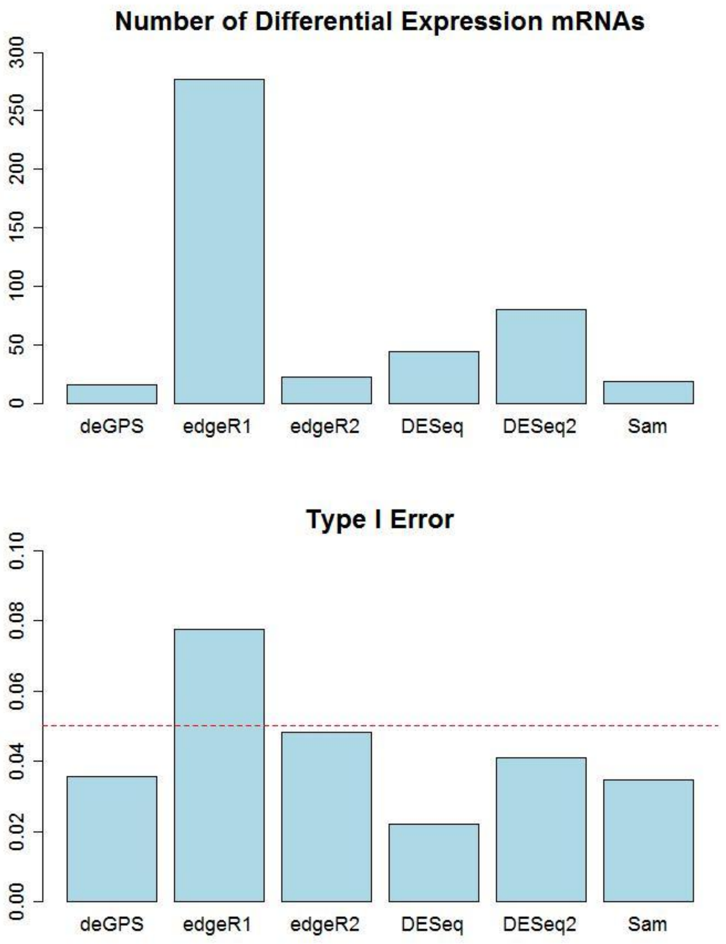

Supplement: Additional file 11: Figure S8. — -Average false positive number and type I error when comparing two groups that are randomly and equally sampled from different developmental stages of Drosophila melanogaster. [file 12864_2015_1676_MOESM11_ESM.pdf]
