# Supplementary material for: deGPS is a powerful tool for detecting differential expression in RNA-sequencing studies
Source: BMC Genomics. 2015 Jun 13;16(1):455. doi: 10.1186/s12864-015-1676-0 (PMC4465298; doi:10.1186/s12864-015-1676-0)

Figure S9. Outliers in Drosophila data. Blue points represent the logarithm of the difference between quantiles and five times sample mean. And red points represent the logarithm of the difference between quantiles and ten times sample mean. compcodeR generates outliers by multiplying the read counts by 5-10. The figure shows that top 2% read counts are larger than 5 times sample mean and top 1% read counts are larger than 10 times sample mean. Though sample mean may not represent the read counts randomly generated by compcodeR properly, we can conclude that up to 1%-2% random outliers are not rare in real data.

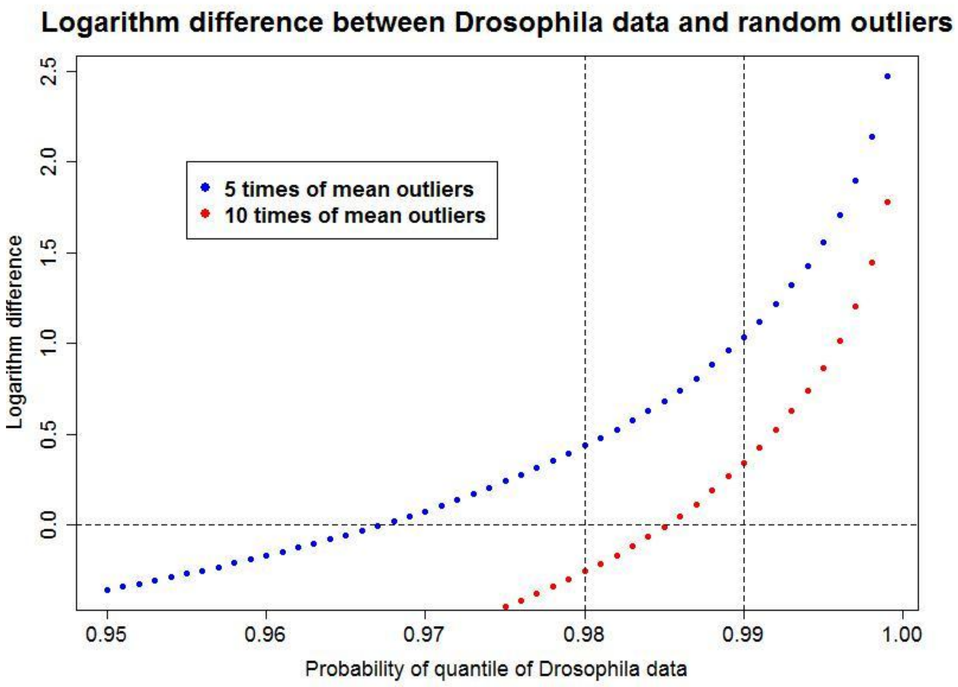

Supplement: Additional file 12: Figure S9. — -Outliers in Drosophila data. Blue points represent the logarithm of the difference between quantiles and five times sample mean. And red points represent the logarithm of the difference between quantiles and ten times sample mean. compcodeR generates outliers by multiplying the read counts by 5–10. The figure shows that top 2 % read counts are larger than 5 times sample mean and top 1 % read counts are larger than 10 times sample mean. Though sample mean may not represent the read counts randomly generated by compcodeR properly, we can conclude that up to 1-2 % random outliers are not rare in real data. [file 12864_2015_1676_MOESM12_ESM.pdf]
