# Supplementary material for: deGPS is a powerful tool for detecting differential expression in RNA-sequencing studies
Source: BMC Genomics. 2015 Jun 13;16(1):455. doi: 10.1186/s12864-015-1676-0 (PMC4465298; doi:10.1186/s12864-015-1676-0)

Figure S10. Overdispersion of sequence count data in RNA-Seq. (**A**) Histogram of $1/(1-\hat{\lambda})$ (in logarithm scale), and (**B**) Sample variance is far away from its mean. Sequence count data were fitted with GP distribution for each sample from TCGA. $1/(1-\hat{\lambda})$ measures the extent of the departure of the data from Poisson distribution.

(A)

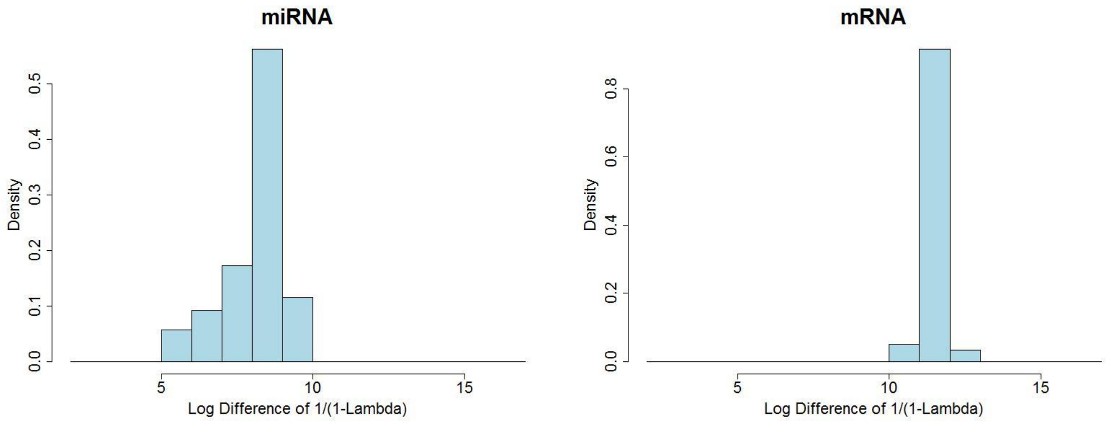

(B)

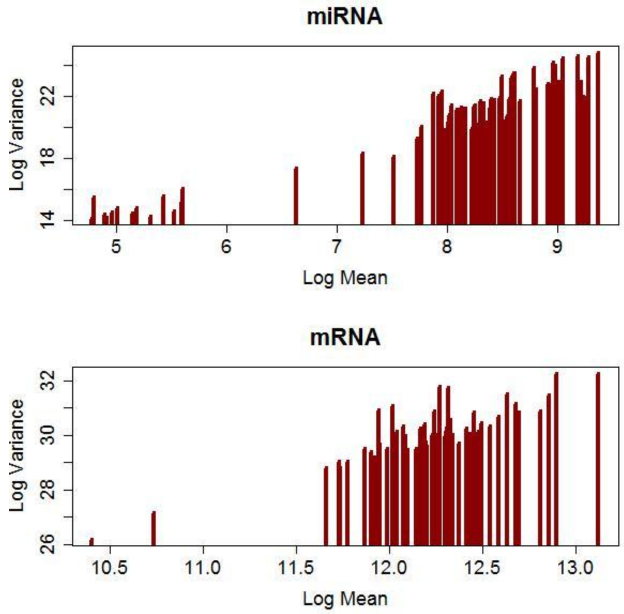

Supplement: Additional file 13: Figure S10. — -Overdispersion of sequence count data in RNA-Seq. (A) Histogram of 1/(1-λ ^) (in logarithm scale), and (B) Sample variance is far away from its mean. Sequence count data were fitted with GP distribution for each sample from TCGA. 1/(1-λ ^) measures the extent of the departure of the data from Poisson distribution. [file 12864_2015_1676_MOESM13_ESM.pdf]
