# Supplementary material for: deGPS is a powerful tool for detecting differential expression in RNA-sequencing studies
Source: BMC Genomics. 2015 Jun 13;16(1):455. doi: 10.1186/s12864-015-1676-0 (PMC4465298; doi:10.1186/s12864-015-1676-0)
Supplement: Additional file 14: Table S3. — -Running times of different R packages for analyzing the development transcriptome of Drosophila. [file 12864_2015_1676_MOESM14_ESM.pdf]

**Table S3.** Running times of different R packages for analyzing the development transcriptome of Drosophila

| deGPS | | DESeq* | DESeq2 | edgeR | SAMseq |
|---|---|---|---|---|---|
| **Number of cores** | **Runtime** | **14 min** | **14 sec** | **10 sec** | **15 sec** |
| **1** | **37.6 min** | | | | |
| **4** | **10.3 min** | | | | |
| **8** | **5.4 min** | | | | |
| **16** | **3.1 min** | | | | |

*Note: DESeq, DESeq2 and edgeR do not use parallel computation and thus one core was used in their analyses.
